# Erythropoietin in tissue engineering and beyond: a multifunctional macromolecule with emerging roles in organoids, immune modulation, and cancer research

**DOI:** 10.3389/fbioe.2026.1708887

**Published:** 2026-06-17

**Authors:** Gaurav Sanghvi, Stefano Bellucci, Suhas Ballal, I. A. Ariffin, Abhayveer Singh, A. Sabarivani, Subhashree Ray, Bhavik Jain, Pankaj Nainwal, A. Deepak

**Affiliations:** 1 Marwadi University Research Center, Department of Microbiology, Faculty of Science, Marwadi University, Rajkot, Gujarat, India; 2 National Institute of Materials Physics, Bucharest-Magurele, Romania; 3 Universidad Ecotec, Samborondón, Ecuador; 4 Department of Chemistry and Biochemistry, School of Sciences, JAIN (Deemed to be University), Bangalore, Karnataka, India; 5 Management and Science University, Shah Alam, Selangor, Malaysia; 6 Centre for Research Impact & Outcome, Chitkara University Institute of Engineering and Technology, Chitkara University, Rajpura, Punjab, India; 7 Department of Biomedical Engineering, Sathyabama Institute of Science and Technology, Chennai, Tamil Nadu, India; 8 Department of Biochemistry, IMS and SUM Hospital, Siksha 'O' Anusandhan (Deemed to be University), Bhubaneswar, Odisha, India; 9 Chitkara Centre for Research and Development, Chitkara University, Baddi, Himachal Pradesh, India; 10 School of Pharmacy, GRD(PG)IMT, Dehradun, Uttarakhand, India; 11 Saveetha Institute of Medical and Technical Sciences, Saveetha School of Engineering, Chennai, Tamil Nadu, India

**Keywords:** cancer, erythropoietin, immobilization, organoids, tissue engineering

## Abstract

Erythropoietin (EPO), a glycoprotein hormone conventionally associated with erythropoiesis, has emerged as a versatile macromolecule with substantial therapeutic potential in tissue engineering and regenerative medicine. Beyond its role in red blood cell production, EPO displays pleiotropic effects, including angiogenesis, neuroprotection, anti-apoptosis, immunomodulation, and cell survival, making it a suitable agent for tissue repair and regeneration. This review explores EPO’s biological characteristics and its integration into tissue-engineered constructs through innovative approaches such as scaffold immobilization, hydrogel encapsulation, and genetically modified cells for localized delivery. EPO has shown remarkable efficacy in regenerating diverse tissues, including bone, cartilage, neural, cardiac, dental, and skin, and in promoting wound healing. Additionally, its applications extend to advanced fields such as organoid development, immune modulation, and cancer research, further highlighting its versatility. Nevertheless, challenges such as maintaining EPO’s bioactivity, achieving controlled and sustained delivery, and mitigating systemic or off-target effects remain significant barriers. Furthermore, its dual role in cancer biology necessitates a deeper understanding of its effects on tumor growth and immunity. Future advances in biomaterials and precision medicine could optimize EPO-based delivery systems to enable personalized therapeutic solutions. EPO stands poised to revolutionize tissue engineering, thus bridging laboratory innovation and clinical applications.

## 1 Introduction

Tissue engineering and regenerative medicine are transformative fields that address critical challenges associated with tissue and organ damage (Pramanik et al., 2023a; Vaidya et al., 2024; Shrivastav, 2022; Yadav et al., 2025a). A key component of tissue engineering involves the development of scaffolds designed to replicate the extracellular matrix (ECM) and thus provide structural and functional support for cellular attachment, proliferation, and differentiation. Polymeric scaffolds are frequently employed due to their biocompatibility and biodegradability. These scaffolds are often fabricated from natural polymers such as collagen, hyaluronic acid, and chitosan, or synthetic materials including polylactic acid, polyglycolic acid, and polycaprolactone (Abourehab, 2022a; Pramanik et al., 2024; Abourehab, 2022b). Despite their versatility, these scaffolds often lack bioactivity, limiting their ability to actively engage in and promote tissue regeneration. This absence of bioactivity poses a significant barrier to achieving optimal outcomes in tissue repair and regeneration. In native tissues, ECM components act as reservoirs for numerous growth factors, cytokines, hormones, and signaling molecules (Boontheekul and Mooney, 2003; Wade and Burdick, 2012; Babensee et al., 2000; Macri et al., 2007; Schultz and Wysocki, 2009) (Taipale and Keski-Oja, 1997). Therefore, an ideal scaffold for tissue engineering should be capable of delivering such signaling molecules in a controlled and sustained manner.

To address the bioactivity limitations of pure polymeric scaffolds, various biochemical cues and drug delivery systems have been incorporated into their structures (Li J. et al., 2021; Bakhs et al., 2023). Techniques include surface modification (Sengupta and V Prasad, 2018), the integration of extracellular matrix components (Valdoz, 2021), and the encapsulation of bioactive molecules (Stratton et al., 2016; Malafaya et al., 2007). Studies have shown that scaffolds modified with small drug molecules (Kyllönen et al., 2015), cytokines (Garg et al., 2011), hormones (Koo et al., 2015), and growth factors (Vija et al., 2019) exhibit significantly higher healing activity than unmodified scaffolds. For instance, growth-factor-loaded wound dressings have demonstrated improved healing potential, highlighting the impact of biochemical augmentation (Losi et al., 2013). Among the numerous bioactive molecules explored for scaffold modification, erythropoietin (EPO) has emerged as particularly promising (Patel et al., 2015).

EPO is primarily produced by the kidneys in response to low oxygen levels; however, during fetal development, the liver is the predominant site of EPO production before the kidneys assume this role after birth. EPO promotes the formation of red blood cells by interacting with specific receptors on immature cells within the bone marrow (Kimáková et al., 2017). Beyond its hematopoietic function, EPO has been found to exhibit an extensive range of non-hematopoietic impacts, including angiogenesis (Kawachi et al., 2012), anti-inflammatory activity (Liu et al., 2015), cell protection (Nasri, 2013), and the modulation of stem cell differentiation (Nair et al., 2013). These properties are especially valuable in tissue engineering, where challenges such as inadequate vascularization, poor cell survival in hypoxic environments, and unregulated inflammatory responses often limit success.

The incorporation of EPO into polymeric scaffolds has improved key aspects of conventional designs by enhancing cellular responses, promoting vascularization, and enabling controlled release for sustained bioactivity. EPO enhances angiogenesis (Yang et al., 2014), which is critical for nutrient delivery and cell survival within the scaffold. Additionally, it protects migrating cells against apoptosis, enhances cell ingrowth, and supports successful scaffold engraftment at injury sites (Vazquez-Mellado et al., 2017). However, EPO’s short half-life (Kontermann, 2016), low stability, and rapid enzymatic degradation present challenges for direct application (Jelkmann et al., 2001). Advanced strategies, such as immobilizing EPO on scaffold surfaces, incorporating it into scaffold matrices, and using EPO-loaded nanoparticles, have been developed to address these issues (Ito et al., 2004; Fayed et al., 2012). These approaches enable spatial and temporal control over the release of EPO, preserving its bioactivity and optimizing therapeutic outcomes. The use of EPO in tissue engineering represents a significant advance in the field, enhancing angiogenesis, protecting cells from oxidative stress and apoptosis, and modulating inflammation to create a conducive microenvironment for tissue repair.

Existing reviews on EPO in tissue engineering have largely emphasized its roles in neuroprotection, angiogenesis, and general regenerative effects. However, critical aspects remain insufficiently explored, particularly its integration into tissue-engineered scaffolds with respect to biomaterial interactions, sustained bioactivity, and delivery strategies. Moreover, mechanistic insights into EPO-mediated stem cell differentiation, extracellular matrix remodeling, and immune modulation are limited. Although its potential in wound healing and bone regeneration has been reported, strategies for controlled release, stability within biomaterial constructs, and translation-related challenges remain inadequately addressed.

A structured literature search was conducted to ensure the comprehensive and transparent inclusion of relevant studies published between 2019 and 2025. Databases including PubMed, Scopus, Web of Science, and Google Scholar were searched using combinations of keywords such as “erythropoietin,” “EPO,” “tissue engineering,” “regenerative medicine,” “organoids,” “immune modulation,” and “cancer research.” Studies were included if they investigated EPO or its derivatives in tissue engineering and related biomedical applications and were published in peer-reviewed English-language journals. Articles lacking relevance or scientific rigor, as well as conference abstracts, editorials, and duplicate publications, were excluded. Selection was based on screening titles, abstracts, and full texts, with emphasis on studies providing strong mechanistic insights and translational relevance.

This review addresses these gaps by analyzing the structural and functional features of EPO, its mechanisms in tissue regeneration, and strategies for its incorporation into biomaterial-based scaffolds. We highlight emerging approaches, including advanced delivery systems, nanocarrier-assisted formulations, and scaffold modifications that enhance therapeutic efficacy. In addition, translational challenges such as immunogenicity, dose optimization, and regulatory considerations are discussed, providing an integrated perspective on the potential of EPO in tissue engineering and regenerative medicine.

## 2 EPO: biological functions and mechanisms

The early understanding of EPO biology was established through the pioneering work of Eugene Goldwasser, whose studies achieved the first successful purification and biochemical characterization of human EPO, laying the foundation for all subsequent mechanistic and translational research in this field (Goldwasser and Kung, 1971). Goldwasser’s subsequent investigations clarified the physiological role of EPO in erythroid differentiation and its regulation by hypoxia, firmly establishing it as a hormone central to red blood cell production rather than a nonspecific humoral factor (Goldwasser, 1975). Building on these foundational discoveries that established EPO as a hypoxia-regulated hormone that drives erythroid differentiation, EPO is a glycoprotein cytokine essential for the regulation of red blood cell production under conditions of low oxygen levels in the blood (Fried, 2009; Jelkmann, 1992). The name “erythropoietin” is derived from the Greek words *erythros* (red) and *poiesis* (to make), reflecting its role in stimulating the production of red blood cells. This cytokine was first identified in the early 20th century during studies on anemia, but its purification and detailed characterization occurred much later, in the 1970s and 1980s, when advances in molecular biology enabled its recombinant production (Jacobs et al., 1985; Lin et al., 1985; Ma et al., 2017). The protein is produced primarily in specialized interstitial fibroblasts located in the kidney’s cortex (Krantz, 1991; Lin et al., 1996) and, to a lesser extent, in the liver, particularly during fetal development. EPO (low molecular weight-30.4 kDa) consists of a 165-amino-acid polypeptide backbone with glycosylation sites that play a crucial role in stabilizing the molecule and extending its circulatory half-life (Ribatti et al., 2007; Jelkmann, 1992). This stability allows EPO to exert its biological effects over a prolonged period, ensuring the effective stimulation of red blood cell precursors. EPO acts by binding to specific receptors known as “EPO receptors,” which are found predominantly on the surface of erythroid progenitor cells in the bone marrow (Mulcahy, 2001). Upon EPO binding, pre-formed receptor dimers undergo a conformational change which activates three major signal transduction pathways (JAK–STAT, PI3K–AKT, and Ras–MAPK). The Janus kinase/signal transducers and activators of transcription (JAK/STAT) pathway facilitate the expression of genes that promote cell survival and proliferation (Lai et al., 2005). Through these pathways, EPO can enhance tumor cell proliferation and confer resistance to apoptosis, particularly under stress conditions such as hypoxia or chemotherapy (Jelkmann, 2011). The phosphatidylinositol 3-kinase and AKT signaling pathway reduces apoptosis by modulating the balance of pro- and anti-apoptotic proteins, while the mitogen-activated protein kinase pathway contributes to cellular differentiation (Ratajczak et al., 2001). Although EPO is traditionally associated with red blood cell production, its roles extend beyond hematopoiesis. EPO has pro-angiogenic effects by directly stimulating endothelial cells and increasing the expression of vascular endothelial growth factor (VEGF), which supports neovascularization within the tumor microenvironment. This enhanced vascular network may facilitate tumor growth and metastasis by improving oxygen and nutrient supply (Wang et al., 2008; Bahlmann et al., 2003). This property makes it particularly valuable in regenerative medicine, where vascularization is a limiting factor in the successful integration of engineered tissues.

EPO is widely recognized for its hematopoietic role, but accumulating evidence highlights its broader regenerative functions, including angiogenesis, neuroprotection, immunomodulation, and cell survival (Watowich, 2011). These pleiotropic effects are mediated by several intracellular signaling cascades. In erythroid progenitors, EPO binds to its receptor, triggering the activation of Janus kinase 2 (JAK2), which subsequently phosphorylates STAT5 (Sawyer and Penta, 1996). Phosphorylated STAT5 dimerizes and translocates to the nucleus, promoting the transcription of anti-apoptotic genes such as Bcl-xL and Bcl-2. Notably, constitutively active STAT5 (cS5) rescues erythropoiesis in EpoR^−/−^ or JAK2^−/−^ cells, enabling CFU-E colony formation and erythroid differentiation independently of EPO signaling (Grebien et al., 2008). This pathway not only sustains erythropoiesis but also contributes to cell survival in various tissues (Ferbeyre and Moriggl, 2011; Paukku and Silvennoinen, 2004). In endothelial cells, EPO stimulates angiogenesis through both direct and indirect mechanisms (Alvarez Arroyo et al., 1998; Haller et al., 1996). Under hypoxic conditions, EPO expression is upregulated by hypoxia-inducible factor-1 alpha (HIF-1α) (Yeo et al., 2008), which also induces VEGF production. EPO further enhances endothelial cell proliferation and migration via the activation of PI3K/AKT and MAPK/ERK signaling pathways. These effects are crucial for neovascularization during tissue repair (Tóthová et al., 2021; Sivertsen et al., 2006). EPO strengthens EC-EC junctions via VE-cadherin and ZO-1 upregulation, reducing vascular leakage in brain and cardiac tissues (Trincavelli et al., 2013). In neural tissue, EPO exhibits neuroprotective activity by reducing apoptosis and oxidative stress, largely through the activation of the JAK2/PI3K/AKT axis and inhibition of caspase pathways, fostering synaptic plasticity. It also modulates excitotoxicity by attenuating glutamate-induced injury and promoting neurotrophic factor expression (Aghoghovwia et al., 2021). Brain pericytes produce EPO under hypoxia, stimulating adjacent ECs to secrete VEGF and SDF-1α, thus promoting neurogenesis and vascular repair (Ji, 2016). Additionally, single-cell transcriptomics reveal that EPO reduces inhibitory interneuron synaptic complexity (e.g., GAD67↓ and GABA receptors↓), disinhibiting pyramidal neurons to enhance cognitive function in schizophrenia models (Curto et al., 2024).

While EPO has demonstrated significant regenerative potential in tissue engineering, its role in cancer progression remains a subject of ongoing debate. EPO exerts pro-angiogenic effects by stimulating endothelial cell proliferation and enhancing vascularization, which is beneficial for tissue regeneration. However, these same properties raise concerns in the context of tumor biology, where excessive angiogenesis can contribute to tumor growth and metastasis. Beyond endothelial cells, EPO directly activates JAK2/STAT3, AKT, and ERK pathways in EPOR-positive cancer cells (e.g., renal, breast, and cervical cancers), upregulating cyclin D1 and suppressing p21/p27 to drive proliferation and chemoresistance (Ribatti, 2009; Pham et al., 2019; Debeljak et al., 2014; Lacombe and Mayeux, 1998). Notably, EPO enhances breast cancer stem cell (BCSC) survival and self-renewal through AKT/ERK activation, increasing resistance to agents such as cisplatin and sunitinib (Vignjević Petrinović et al., 2022). Paradoxically, EPO also interacts with alternative receptors such as EphB4 in ovarian and breast cancers, activating STAT3 to promote tumor growth independently of canonical EPO receptors (Pradeep et al., 2015). Clinically, recombinant EPO (rhEPO) use correlates with increased mortality in cancer patients due to tumor progression and metastasis, particularly in EPOR-expressing malignancies (Phillips et al., 2007; Baz et al., 2006), (Lönnroth et al., 2008).

The dual nature of EPO presents a challenge in biomedical applications. While it enhances wound healing and tissue repair, its potential to support malignant cell survival necessitates caution. EPO stimulates tumor angiogenesis via VEGF-independent pathways by activating EPOR signaling in endothelial cells, increasing micro-vessel density, and enhancing neovascularization, as shown in mammary carcinoma models where EPO blockade reduced angiogenesis by 60% (Debeljak et al., 2014; Hardee et al., 2007; Annese et al., 2019). To mitigate these risks, strategies such as localized and controlled EPO delivery within biomaterial scaffolds have been explored. Encapsulation within hydrogels, nanoparticle-based carriers, and scaffold modifications that regulate EPO’s release kinetics can help maintain therapeutic efficacy while minimizing systemic exposure. Other strategies include EPOR antagonists (e.g., R103A-EPO), EphB4-targeted therapies, localized biomaterial-based delivery to minimize systemic exposure, employing companion diagnostics (e.g., EpoR-Cy5.5 probes) to ensure EPO doses remodel vasculature without reaching pro-survival thresholds in cancer cells, and combinatorial approaches with PARP inhibitors to counteract EPO-induced survival pathways (Liu et al., 2013; McKinney and Arcasoy, 2011; Inbar et al., 2012; Doleschel et al., 2015). Prolonged EPO treatment in patients with pre-existing malignancies also poses potential risks, as EPO may promote tumor progression by enhancing angiogenesis, supporting cell survival, and reducing apoptosis. A recent study in a murine breast cancer model demonstrated that prolonged EPO treatment promoted tumor growth by inducing an immunosuppressive tumor microenvironment, accelerating CD4^+^ T cell exhaustion, and increasing CD39^+^ regulatory T cell populations (Bessoles et al., 2024). Tumors expressing EPOR can respond to EPO signaling, potentially leading to increased proliferation and therapy resistance. Some clinical studies have linked EPO use in cancer patients to worsened outcomes, prompting caution in its application. Therefore, while EPO offers therapeutic benefits, its use in oncology requires careful risk–benefit assessment.

EPO also plays a critical role in cellular environments exposed to hypoxic stress, particularly in the kidney, where impaired oxygen sensing and reduced EPO production lead to diminished erythroid progenitor survival and anemia, underscoring the essential role of EPO in maintaining erythropoietic homeostasis under stress conditions (Brines and Cerami, 2008; Ghezzi and Brines, 2004; Eschbach et al., 1989). Additionally, EPO exerts immunomodulatory effects by suppressing pro-inflammatory cytokines such as TNF-α, IL-1β, and IL-6 while enhancing IL-10 levels, often via the downregulation of the NF-κB signaling pathway. These immunomodulatory actions are complemented by EPO’s influence on macrophage polarization, encouraging a shift toward the reparative M2 phenotype (Nairz et al., 2012; Jelkmann, 2011). Another noteworthy function of EPO is its influence on stem cell biology. It enhances the survival and proliferation of stem cells, particularly mesenchymal stem cells (MSCs), which are critical for tissue regeneration. EPO also guides the differentiation of these cells into specific lineages, such as bone-forming osteoblasts or cartilage-producing chondrocytes, making it highly versatile for use in repairing specialized tissues. In bone regeneration, EPO has been shown to support the osteogenic differentiation of MSCs through the activation of BMP/Smad and Wnt/β-catenin pathways (Li W. et al., 2021). It also upregulates stromal-derived factor-1 (SDF-1) and its receptor CXCR4, facilitating the targeted migration of MSCs to sites of injury. This chemotactic effect enhances local cell availability for bone repair (Lacombe and Mayeux, 1999; Wu et al., 1995; Jelkmann, 2004).

The production of EPO is tightly regulated to maintain oxygen homeostasis in the body, with hypoxia serving as the primary stimulus. Under low oxygen levels, specialized interstitial fibroblasts in the renal cortex, and to a lesser extent in the liver, upregulate EPO synthesis (Bunn, 2013). This regulation is mediated by hypoxia-inducible factor 1-alpha (HIF-1α), a transcription factor stabilized during hypoxic conditions. HIF-1α dimerizes with HIF-1β and binds to hypoxia-response elements in the EPO gene promoter, driving its transcription (Haase, 2013; Stockmann and Fandrey, 2006). Conversely, in normoxic conditions, HIF-1α is hydroxylated by prolyl hydroxylase enzymes, marking it for degradation via the ubiquitin–proteasome pathway, thereby suppressing EPO production (Kang et al., 2005). The feedback mechanism plays a critical role in EPO regulation. Increased red blood cell mass or improved oxygen delivery reduce hypoxic stimuli, lowering EPO synthesis (Moritz et al., 1997; Ifeanyi, 2015). This negative feedback ensures that erythropoiesis remains balanced and prevents conditions such as erythrocytosis. Other factors influencing EPO regulation include inflammatory cytokines such as interleukin-1 (Frede et al., 1997) and tumor necrosis factor-alpha (Buck et al., 2008), which suppress its production, contributing to anemia in chronic disease. Conversely, hormones such as androgens and insulin-like growth factor 1 (IGF-1) can enhance EPO synthesis (Kadri, 2015). Additionally, nitric oxide and reactive oxygen species generated during oxidative stress have been shown to modulate EPO expression, though their effects can vary depending on the context (Daghman et al., 1999). The liver plays a dominant role in EPO production during fetal development, while the kidney becomes the primary postnatal source. This transition reflects the changing physiological demands and organ functionality during development. Research has revealed that VEGF acts as a negative regulator of hepatic EPO synthesis through VEGFR2-dependent paracrine signaling, with its inhibition leading to significant increases in hepatic EPO production and erythrocytosis, offering insights into alternative regulatory mechanisms of erythropoiesis (Tam et al., 2006).

Despite its therapeutic potential, the application of EPO in clinical settings faces significant challenges. Its relatively short half-life in circulation, typically 4–6 h, requires repeated administration for sustained efficacy (Egrie et al., 1993). Moreover, the molecule is rapidly degraded by enzymes, reducing its functional activity (Gross and Lodish, 2006). Achieving therapeutic concentrations through systemic administration can result in side effects such as increased blood viscosity (Singh et al., 1993) and a higher risk of thromboembolic events due to excessive red blood cell production. To overcome these limitations, researchers have developed advanced delivery systems to enhance the efficacy and stability of EPO (Ishii et al., 2012). Encapsulation within biodegradable nanoparticles or hydrogels protects the molecule from enzymatic degradation while enabling controlled release over time. Immobilization onto the surface of biomaterials, such as scaffolds, allows for localized delivery and preservation of activity, reducing the need for systemic exposure (Orive et al., 2005; Murua et al., 2007). These innovations have significantly improved the utility of EPO in tissue engineering and regenerative medicine.

**FIGURE 1:**
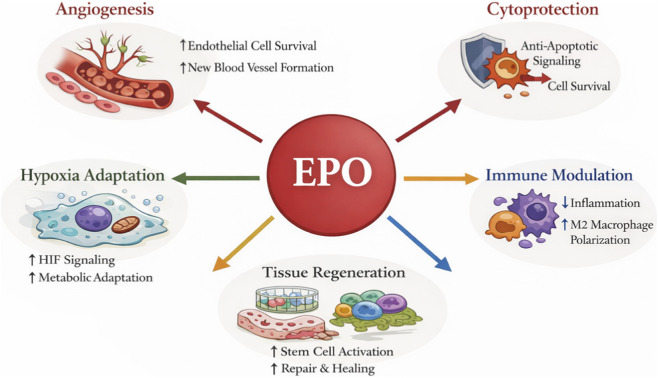
Pleiotropic biological functions of erythropoietin relevant to tissue engineering and emerging applications.

EPO represents a unique macromolecule with multifaceted biological functions that extend far beyond its role in stimulating red blood cell production. Its capacity to promote angiogenesis, protect cells under stress, regulate immune responses, and influence stem cell behavior highlights its value in tissue engineering applications. The diverse, context-dependent biological functions of EPO relevant to tissue engineering and emerging applications are summarized in Figure 1. By addressing the challenges associated with its use, EPO can be further optimized to contribute to the development of advanced therapeutic strategies for tissue repair and regeneration.

## 3 Strategies for incorporating EPO into tissue-engineered constructs

**FIGURE 2:**
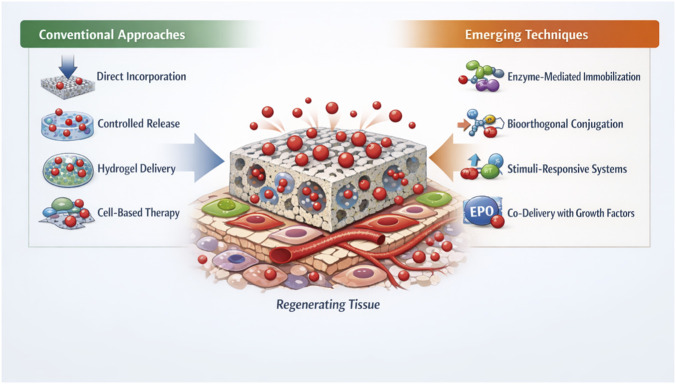
Schematic overview of conventional and emerging strategies for incorporating EPO into tissue-engineered constructs.

The incorporation of EPO into scaffolds requires a range of immobilization and delivery strategies to preserve its bioactivity, control its release, and ensure localized delivery at the site of tissue repair. These methods address challenges such as EPO’s short half-life, enzymatic degradation, and potential systemic side effects. Figure 2 illustrates the various strategies for incorporating EPO into tissue-engineered constructs, highlighting both conventional delivery approaches and emerging immobilization techniques. Below is a comprehensive discussion of the primary immobilization and incorporation techniques used in tissue-engineered constructs.

**FIGURE 3:**
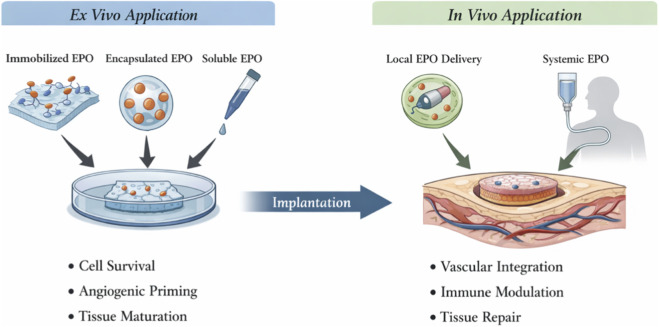
Schematic illustrating the timing and mode of EPO application in tissue engineering, distinguishing between *ex vivo* incorporation during construct preparation and *in vivo* administration following implantation, along with associated biological outcomes.

In tissue engineering applications, EPO can be administered at different stages of construct development, with important implications for biological outcomes and translational feasibility. EPO may be incorporated *ex vivo* during the preparation of engineered tissues, for example, through scaffold immobilization, encapsulation within delivery systems, or supplementation of culture media, to enhance cell survival, angiogenic priming, and tissue maturation prior to implantation. Alternatively, EPO can be delivered *in vivo* following implantation, either locally at the implant site or systemically, to promote vascular integration, modulate immune responses, and support tissue repair. The timing and mode of EPO application therefore represent key design parameters in the development of EPO-functionalized tissue-engineered constructs (Figure 3).

One of the most common approaches is physical encapsulation (Chen et al., 2010), where EPO is embedded within the scaffold matrix during its fabrication. This technique allows the cytokine to be evenly distributed throughout the material and released gradually as the scaffold degrades. The benefit of this approach lies in its ability to maintain the optimized physicochemical properties of the scaffolds while effectively preserving the bioactivity of the loaded bioactive peptides. Methods such as electrospinning are widely used, as they enable the formation of nanofibrous scaffolds with a high surface-area-to-volume ratio, facilitating controlled release (Naseri-Nosar et al., 2017), (Goonoo, 2017). Other fabrication techniques, such as freeze-drying (Gurruchaga et al., 2015) and solvent casting (Senatov et al., 2022), also effectively incorporate EPO into porous scaffolds. While physical encapsulation protects EPO from enzymatic degradation, its main limitations include potential loss of bioactivity during processing and challenges in fine-tuning the release profile. Shi et al. (2012) demonstrated that recombinant human EPO (rhEPO) could be effectively encapsulated into PEG–PLA micelles with high encapsulation efficiency (up to 80%), significantly enhancing its stability and plasma retention time while maintaining bioactivity, as evidenced by increased hemoglobin concentrations in pharmacokinetic studies. Similarly, Hirakura et al. (2010) demonstrated the development of a hybrid hyaluronan–hydrogel system incorporating cholesteryl pullulan (CHP) nanogels, which successfully encapsulated therapeutic proteins such as EPO, enabling sustained release and preserving protein stability through molecular chaperone-like activity. Chow et al. (2017) demonstrated that EPO encapsulated within a polyethylene glycol hydrogel significantly improved cardiac function post-myocardial infarction by increasing infarct thickness and muscle content, thus emphasizing the potential of bioactive hydrogels in regenerative cardiac therapies.

Physical adsorption is another widely used method that relies on non-covalent interactions, such as electrostatic forces, hydrogen bonding, and van der Waals interactions, to bind EPO to scaffold surfaces (Karpov et al., 2020; King and Krebsbach, 2012). This technique is straightforward and preserves EPO’s structural integrity. Scaffold surfaces are often pretreated with plasma or chemical agents to enhance surface charge or roughness, thus increasing adsorption efficiency. While this method effectively maintains the structural integrity and functionality of bioactive peptides, its primary limitation lies in its low loading capacity, allowing only minimal amounts of peptides to be incorporated into scaffolds, often resulting in an initial burst release of the drug. In addition, the weak binding forces involved in physical adsorption can result in rapid desorption under dynamic conditions, such as blood flow or mechanical stress, limiting its long-term efficacy (Jaiswal et al., 2013). Saenz del Burgo et al. (2017) revealed that graphene oxide (GO) nanoparticles can effectively adsorb therapeutic proteins such as EPO on their surface, with the formation of a protein biocorona further enhancing the release profile and functionality of EPO in hybrid alginate–GO microcapsules, which were shown to be effective *in vivo*. Ciriza et al. (2018) demonstrated that incorporating protein-coated graphene oxide (GO) into hybrid alginate microcapsules significantly enhanced the survival of genetically engineered cells that secrete EPO and improved sustained protein release, with larger microcapsule diameters further optimizing therapeutic outcomes *in vitro* and *in vivo.* Boggione et al. (2018) demonstrated that chemically modified chitosan matrices, such as CHS–EPO and CHS–GLUT, exhibited enhanced adsorption properties due to increased surface roughness and effective chemical functionalization, highlighting their potential for bioseparation processes and their applicability in systems involving bioactive molecules such as EPO.

Covalent immobilization provides a more robust solution by forming strong chemical bonds between cytokines and functional groups on scaffold surfaces (Recker et al., 2011). Carbodiimide chemistry, using agents such as EDC and NHS, is one of the most commonly employed methods for this (Köwitsch et al., 2011). This technique facilitates bonding between carboxyl groups on scaffold materials and amine groups on EPO, ensuring stable attachment and prolonged retention. It addresses the issue of burst release associated with bioactive peptides. This approach has proven to be an effective and dependable method for maintaining the stability of bioactive peptides and ensuring their prolonged retention on scaffolds (De Witte et al., 2020). Other covalent methods include polydopamine coatings (Yang et al., 2012), which create a bioinspired adhesive layer that can securely bind EPO, and bio-orthogonal click chemistry (Azagarsamy and Anseth, 2013), which enables site-specific conjugation without interfering with EPO’s functional domains. Polydopamine layers can be effortlessly applied to a variety of surfaces, irrespective of their surface chemistry, and create regions with reversible, noncovalent interactions that exhibit strong affinity and excellent stability. Chen et al. (2018) demonstrated that polydopamine-coated magnetic nanoparticles (MNP@PDA) provide a robust platform for EPO immobilization, utilizing strong covalent-like and affinity-based interactions to ensure stability and enhanced functionality. They highlighted the versatility of MNP@PDA in biosensing and separation, emphasizing its potential for therapeutic applications, including scaffold-based EPO delivery systems. While covalent immobilization ensures strong and stable binding, the process must be carefully optimized to prevent EPO denaturation during chemical modification.

Heparin-based immobilization is another effective method for integrating EPO (Gore et al., 2014). Heparin, known for its high affinity for growth factors (Capila and Linhardt, 2002), stabilizes and localizes EPO on scaffolds. By coating scaffold surfaces with heparin or embedding it within the scaffold matrix, EPO can be bound via electrostatic interactions. This approach not only enhances the stability of EPO but also synergizes with its proangiogenic effects, promoting vascularization in tissue-engineered constructs. Excessive use of heparin, however, may alter the scaffold’s mechanical properties and must be carefully controlled (Rohman et al., 2009). Karyagina et al. (2018) demonstrated that recombinant EPO with an added heparin-binding protein domain (HBD-EPO) could reversibly bind to demineralized bone matrix (DBM), mimicking heparin-based immobilization mechanisms. This approach effectively localized HBD-EPO at the DBM site, enabling it to retain angiogenic activity and support bone regeneration.

Nanoparticle-aided immobilization is a versatile and advanced approach for integrating bioactive molecules such as EPO into delivery systems, providing both protection and controlled release capabilities (Pramanik et al., 2021). Nanoparticles, ranging from polymeric materials such as PLGA and chitosan to inorganic options such as silica and gold, offer a high surface area and modifiable properties for efficient immobilization (Zhang and Uludağ, 2009). These nanoparticles can encapsulate EPO via techniques such as emulsion-based encapsulation or ionic gelation to ensure the stability of the protein while safeguarding it from enzymatic degradation (Venkatesan et al., 2005; Hajimiri et al., 2015). Functionalized nanoparticles further enhance the immobilization process by incorporating ligands, such as targeting moieties or bioactive coatings such as polydopamine, which strengthen the interaction with EPO and enable site-specific delivery. In addition, release profiles can be tailored based on environmental triggers, such as pH changes, enzymatic activity (ter Horst et al., 2016), or the degradation of the nanoparticle matrix, ensuring precise and sustained therapeutic effects (Goldring and Goldring, 2004). This approach is particularly beneficial for localized delivery, reducing systemic exposure and minimizing side effects. Despite its advantages, challenges such as scalability, nanoparticle stability, and potential cytotoxicity need to be addressed. Nonetheless, nanoparticle-aided immobilization continues to be a cornerstone in regenerative medicine and therapeutic delivery, enabling innovative strategies for EPO utilization and other bioactive molecules. Xue et al. (2020) demonstrated that rh-EPO encapsulated in Tween 80-modified albumin nanoparticles could enhance its transport across the blood–brain barrier, enabling localized delivery and superior therapeutic effects for traumatic brain injury treatment. Similarly, Bulmer et al. (2012) revealed that rHu-EPO encapsulated in chitosan-tripolyphosphate nanoparticles, produced via ionotropic gelation, achieved controlled release over 2 weeks, highlighting their potential for prolonged therapeutic applications.

Emerging techniques, such as enzyme-mediated immobilization (Homaei et al., 2013) and bio-orthogonal conjugation methods (Pei, 2022; Scinto et al., 2021; Guo et al., 2017), offer innovative solutions for EPO integration. Enzyme-mediated approaches, such as transglutaminase crosslinking, facilitate the selective and stable attachment of EPO to scaffold surfaces. Bio-orthogonal click chemistry provides high specificity and minimal disruption to EPO’s structure, making it particularly suitable for multifunctional scaffolds. These techniques are highly adaptable and can be tailored to specific applications, although their complexity and cost remain limiting factors for widespread adoption (Ramil and Lin, 2013).

Each immobilization strategy offers unique advantages and challenges, making the choice of technique dependent on the specific requirements of the application. While physical methods prioritize simplicity and cost-effectiveness, chemical and advanced approaches provide greater control, stability, and precision. The integration of EPO into tissue-engineered constructs is a critical step toward enhancing their regenerative potential, paving the way for advanced therapies in regenerative medicine. Addressing the challenges associated with each method and optimizing their implementation will be essential for maximizing the therapeutic benefits of EPO.

## 4 Innovative roles of EPO-modified constructs in tissue repair

**FIGURE 4:**
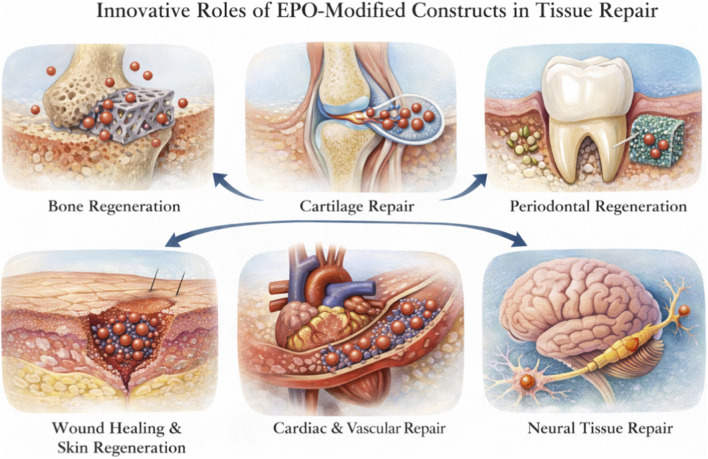
Overview of the regenerative applications of EPO-modified constructs, illustrating their roles in bone, cartilage, periodontal, wound and skin, cardio-vascular, and neural tissue repair through enhanced cell survival, angiogenesis, and tissue regeneration.

EPO-modified constructs have emerged as powerful tools for enhancing regenerative medicine outcomes. EPO-modified constructs have been widely explored to enhance bone, cartilage, periodontal, wound and skin, cardio-vascular, and neural tissue repair by promoting angiogenesis, cell survival, and regenerative signaling (Figure 4).

### 4.1 Bone regeneration

Bone regeneration is a critical area in tissue engineering; it addresses challenges such as poor vascularization and delayed healing in large defects (Abourehab, 2022c; Ab and ourehab, 2022). EPO-modified constructs enhance both angiogenesis (Holstein et al., 2011) and osteogenesis by recruiting endothelial cells and promoting mesenchymal stem cell (MSC) differentiation into osteoblasts (Rö et al., 2012; Wan et al., 2014). For instance, scaffolds composed of hydroxyapatite or β-tricalcium phosphate infused with EPO have shown accelerated mineralization and vascular infiltration, making them ideal for treating critical-sized defects (Senatov et al., 2020). In preclinical models, combining EPO with bone morphogenetic proteins (BMPs) has demonstrated synergistic effects, enhancing new bone formation and vascular development (Shiozawa et al., 2010). These constructs are being developed for non-union fractures, spinal fusion surgeries, and craniofacial reconstructions, thus offering hope for patients with complex orthopedic conditions.

**FIGURE 5:**
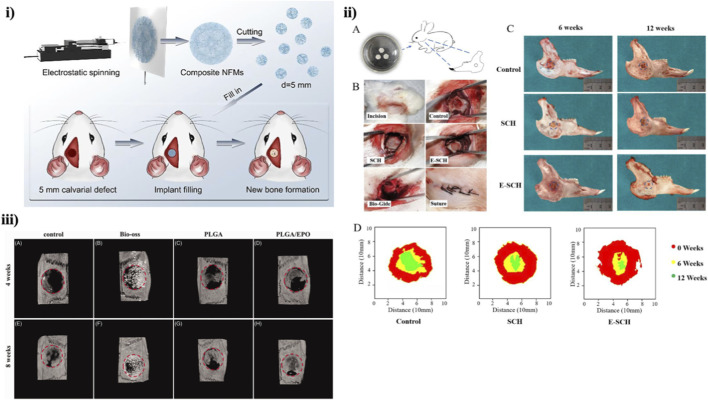
**(i)** Composite nanofiber membranes based on Type-I collagen polypeptides for rapid and effective bone regeneration , reproduced with permission from Jin et al. (2024) (© 2024, ACS). **(ii)** Mandibular defect and *in vivo* reconstruction. **(A)** Diagrammatic representation of the study design. **(B)** Process for preparing the rabbit mandibular defect model. **(C)** Reconstructed mandibles collected at different time points. **(D)** Diagrammatic depiction of the bone defect healing process at 0, 6, and 12 weeks post-surgery, reproduced with permission from Liu et al. (2022) (© 2022, ACS). **(iii)** 3D Micro-CT images showcasing rat calvarial defect sites at 4 and 8 weeks post-implantation. The images represent four groups: control, Bio-Oss, PLGA, and PLGA/EPO. The defect areas are highlighted with red circles (Yu et al., 2020).

Jin et al. (2024) introduced a novel strategy for bone regeneration by designing hydroxyapatite, EPO, and osteogenic growth peptide (OGP) co-doped type-I collagen (Col I) polypeptide nanofiber membranes (NFMs) using electrospinning technology (Figure 5i). This scaffold-based defect platform provides a relevant environment to assess EPO’s role in enhancing BMSC recruitment, osteogenic differentiation, and vascularization. Given the known pro-osteogenic and angiogenic properties of EPO, its integration into such models has been shown to significantly improve bone healing outcomes, further supporting its therapeutic potential in bone tissue engineering. This composite scaffold demonstrated exceptional biocompatibility and osteogenic potential. *In vitro* studies revealed low cytotoxicity and enhanced osteogenic differentiation of rat bone marrow mesenchymal stem cells (BMSCs), evidenced by an elevated expression of osteogenic genes, strong alkaline phosphatase activity, and calcium nodule formation confirmed by alizarin red S staining. *In vivo* experiments showed that hydroxyapatite established mineralization centers within the defect site, while Col I, EPO, and OGP synergistically promoted bone growth along these centers to achieve complete bone regeneration within 2 months. Hematological and histological assessments verified the safety and efficacy of the scaffold. The limitations of Jin et al. (2024) include uncertain long-term stability, reliance on a single animal model, limited insight into immune interactions, and unexamined mechanical resilience. Future research should address these gaps for better clinical translation.

Vasileva et al. (2024) investigated EPO’s dual role in orthopedics, emphasizing its angiogenic and osteoinductive properties in bone regeneration. They assessed bone turnover markers (BALP, PINP, CTX, and DPD) during femoral fracture healing in cats treated with plate osteosynthesis. EPO-treated cats exhibited earlier peaks in BALP and PINP by the second week post-surgery, indicating accelerated bone formation, while bone resorption markers remained unaffected. Their study is limited by its small sample size, short 2-month observation period, and lack of consideration for age, sex, or breed variations. They focused on a limited set of bone markers and did not explore long-term effects or underlying mechanisms, highlighting the need for broader, more detailed research. Future studies should focus on EPO-integrated biomaterial scaffolds, optimized delivery systems, and long-term clinical evaluation.

Salehi et al. (2021) introduced a novel cell-free scaffold for bone tissue engineering and utilized EPO to enhance localized bone regeneration while addressing systemic side effects. The polylactic acid/nanoclay/nanohydroxyapatite scaffold, fabricated using thermally induced phase separation, demonstrated sustained EPO release, favorable physical properties, and biocompatibility with MG-63 osteoblast-like cells. Using a rat calvarial defect model, they demonstrated the efficacy of an EPO-containing scaffold in promoting bone regeneration. After 8 weeks, the scaffold facilitated substantial bone formation, with approximately 41% of the defect area filled with new bone tissue. Additionally, they observed increased vascularization and the presence of osteoblasts in the regenerated area. Despite promising *in vitro* and *in vivo* outcomes, Salehi et al. (2021) had some limitations. The initial burst release of EPO (∼30% in the first 36 h) may reduce long-term effectiveness. The duration of sustained EPO release was limited to 2 weeks, which may not be sufficient for complete bone regeneration. Additionally, they used a single EPO dosage (3750 units) without dose-response optimization. Future research should optimize scaffold properties, validate findings in larger animal models, and explore synergistic combinations of EPO with other growth factors for enhanced therapeutic outcomes to advance this approach toward clinical applications in regenerative medicine. Moreover, further studies using advanced markers and molecular analyses are required to understand the exact mechanisms of MSC recruitment, angiogenesis, and bone remodeling.

Liu et al. (2022) developed a 3D-printed composite scaffold (SCH) that incorporated silk fibroin, collagen, and hydroxyapatite for localized EPO delivery in bone regeneration. The low-temperature fabrication preserved biomaterial integrity, enabling sustained rh-EPO release. The resulting E-SCH scaffolds exhibited ideal porosity (89.4% ± 3.2%), high water absorption (∼979%), and a sustained rh-EPO release profile, with 63.1% released in 12 h and 71.6% in 96 h. *In vitro*, EPO incorporation promoted MC3T3-E1 cell proliferation (*p* < 0.05 on days 5 and 7) without affecting scaffold mechanics (elastic modulus ∼40 kPa). *In vivo* rabbit studies demonstrated scaffold degradation, enhanced osteoblast activity, collagen fiber formation, and mandibular defect reconstruction. Figures 5iiA–D illustrate the experimental setup, surgical model, defect healing trajectory, and comparative healing outcomes. By week 12, the E-SCH group showed the most advanced bone regeneration with no inflammation. Although the E-SCH scaffolds demonstrated promising *in vitro* and *in vivo* performance, certain limitations remain. The study was conducted in a small animal model (rabbits), which may not fully replicate clinical conditions in humans. Additionally, the observation period was limited to 12 weeks, which may not capture long-term effects such as complete scaffold degradation and mature bone remodeling. The release profile of rh-EPO showed a significant initial burst, which might reduce its sustained osteogenic and angiogenic effects. Furthermore, Liu et al. (2022) did not explore varying doses of rh-EPO or compare the scaffold’s performance against other clinically used materials in detail. Future research should focus on optimizing scaffold designs for patient-specific applications, evaluating long-term efficacy in larger models, and integrating additional growth factors for enhanced regeneration.

Recent studies, including Wang et al. (2022), highlight EPO’s dual capacity to stimulate both bone formation and blood vessel growth, emphasizing its potential in regenerative medicine. In Wang et al. (2022), biphasic calcium phosphate was functionalized with polydopamine and EPO, enabling sustained EPO release. Co-cultures of rat vein endothelial cells (VECs) and BMSCs demonstrated enhanced cell proliferation, upregulated osteoblastic and endothelial markers, and increased EphB4/EphrinB2 expression. Implantation in Sprague–Dawley rat femurs showed effective bone defect repair with significant upregulation of EphB4/EphrinB2 signaling, highlighting its critical role. Despite promising results, their study has some limitations. It was conducted on a small animal model (rats), which may not fully represent human clinical outcomes. The release of EPO, while improved with polydopamine coating, still showed a relatively short release period and may require further *in vivo* confirmation for long-term effectiveness. Additionally, while the EphB4/EphrinB2 signaling pathway was identified as a key mechanism, they did not explore the detailed molecular crosstalk between this and other pathways involved in bone regeneration. The long-term biocompatibility and degradation behavior of the modified bioceramics were also not addressed in depth, suggesting the need for extended follow-up and testing in larger animal models or clinical settings. Future work will focus on optimizing the release kinetics, exploring alternative bioceramic carriers and investigating translational potential in clinical models.

Critical-size bone defect models are pivotal for assessing the osteogenic potential of biomaterials. Vasileva and Chaprazov (2023) evaluated recombinant human EPO for trabecular bone healing in a rat femoral critical-size defect model. They assigned 56 Wistar albino rats to six groups: a control group (untreated defects) and five experimental groups treated with absorbable collagen cones soaked in saline, EPO, or EPO combined with a xenograft, plus a group receiving systemic subcutaneous EPO. Bone regeneration was assessed via radiography, osteodensitometry, and histology at 30 and 90 days. Results showed that local EPO on a collagen scaffold significantly enhanced bone healing, while systemic EPO had limited impact. Combining EPO with a xenograft further improved graft–host integration. Vasileva and Chaprazov (2023) used a single high systemic dose of EPO, which showed limited effectiveness compared to repeated dosing protocols in other studies. The sample size across groups was relatively small, and outcomes were evaluated only up to 90 days, limiting long-term insight. While radiological, densitometric, and histological analyses were performed, they did not explore the molecular mechanisms behind EPO-induced bone regeneration. Broader studies with varied dosing, extended timelines, and mechanistic analyses are needed for clinical translation.

Building on the exploration of EPO’s therapeutic potential in bone regeneration, Chaprazov et al. (2022) compared local and systemic applications of rhEPO for healing rat calvarial defects. EPO, primarily recognized for its erythropoiesis-stimulating effects, also exhibits diverse bone-related functions. These include enhancing osteoblast growth, fostering blood vessel formation, accelerating early bone development through cartilage ossification, and boosting bone mechanical properties. Systemic effects of rhEPO were evaluated through hematological parameters—erythrocyte count, hemoglobin, and hematocrit—at 0, 30, and 90 days post-administration, while bone regeneration was assessed via radiographic and computed tomography imaging. The results indicated that intraperitoneal rhEPO administration, while effective in stimulating erythropoiesis, failed to enhance bone formation. In contrast, a single local application of rhEPO that employed a collagen carrier greatly improved bone healing without causing systemic side effects. Chaprazov et al. (2022) were limited by their small sample size, short duration, and use of a single high systemic EPO dose, which may not reflect optimal treatment protocols. They lacked histological or molecular analysis to clarify mechanisms and relied on imaging alone to assess bone regeneration. Their findings need validation through longer-term studies with varied dosing and larger models. Future directions include optimizing carrier materials, refining dosing regimens, and exploring potential synergies with other regenerative therapies to maximize clinical utility.

Lafuente-Merchan et al. (2022) investigated the potential of 3D bioprinting for addressing bone damage caused by trauma, tumors, and aging-related diseases such as osteoporosis. They enhanced nanocellulose–alginate (NC–Alg)-based bioink with hydroxyapatite (HAP) and graphene oxide (GO) to improve its properties. Rheological behavior, biocompatibility, and the effects of sterilization were evaluated, followed by scaffold fabrication and biological tests using murine D1 mesenchymal stem cells engineered to produce EPO. HAP and GO preserved bioink viscosity during sterilization, but GO was degraded by short-cycle autoclaving, limiting its compatibility. Both components improved scaffold mechanics, cell viability, and functionality, with GO showing superior bone differentiation potential. GO scaffolds also exhibited the highest Young’s modulus, improving swelling (∼90%). HAP-containing scaffolds showed significantly higher EPO release (1483.90 mLUI/mL at day 21) than GO (337.93 mLUI/mL) and control (925.21 mLUI/mL) scaffolds. Their study is limited to *in vitro* experiments, with no *in vivo* validation to confirm the scaffold’s long-term performance in actual bone regeneration. The autoclave sterilization process altered the chemical structure of GO, potentially increasing cytotoxicity and limiting its use. Additionally, there was a delay in EPO release from GO-containing scaffolds, likely due to EPO binding to GO, which could reduce therapeutic effectiveness. Lafuente-Merchan et al. (2022) also used fixed concentrations of GO and HAP without exploring dose optimization. Furthermore, while mechanical properties were improved, they remained significantly lower than those of native bone, suggesting the scaffolds may be better suited for non-load-bearing applications unless further reinforced. Future research should focus on optimizing sterilization methods and exploring clinical applications.

Extending the discussion on EPO’s influence on bone metabolism, Awida et al. (2022) shifted focus to the contrasting roles of its receptor forms. While the homodimeric EPO receptor (EPOR) governs hematopoiesis, tissue protection is mediated by a heteromeric receptor comprising EPOR and CD131. EPO’s promotion of osteoclast precursors and subsequent bone loss is recognized, yet the mechanisms remain unresolved. They utilized cibinetide (CIB), a selective non-erythropoietic EPO analogue that targets the heteromeric receptor to examine its effects. Female C57BL/6J WT mice treated with CIB for 1 month showed marked improvements in bone mineral density—∼5.8% in cortical bone and ∼5.2% in trabecular bone. Flow cytometry analysis revealed that the combination of CIB and EPO, administered over a 5-day period, with EPO given on first and fourth day, resulted in a significant 42.8% reduction in osteoclast precursor cells. *In vitro* studies further confirmed that CIB, either alone or paired with EPO, effectively inhibited osteoclastogenesis. The main limitation of this study is its reliance on short-term treatment regimens, which may not fully capture the long-term effects of CIB on bone remodeling and microarchitecture; additionally, the molecular mechanisms by which CIB exerts opposing effects to EPO via the EPOR/CD131 heteroreceptor remain unclear, and the absence of conditional knockout models for CD131 in osteoblasts and osteoclasts limits mechanistic insights into cell-specific actions. The results obtained by Awida et al. (2022) highlight CIB’s potential to mitigate EPO-associated bone loss, offering a promising direction for future therapeutic development and clinical application in bone preservation strategies.

EPO plays a crucial role in bone defect repair by promoting osteogenesis and angiogenesis, particularly under hypoxic conditions. Yu et al. (2020) demonstrated that rhEPO enhances endothelial progenitor (EPC) and marrow stromal (ST2) cell proliferation without inducing apoptosis, upregulating angiogenic and osteogenic factors. In a rat calvaria defect model, PLGA scaffolds encapsulating rhEPO significantly accelerated bone formation, as confirmed by Micro-CT analysis (Figure 5iii). At 4 weeks, minimal new bone formation was observed in the control group (Figure 5iiiiA), while Bio-Oss-treated defects contained dispersed material (Figure 5iiiB). In contrast, PLGA scaffolds supported new bone formation (Figure 5iiiC, D), with rhEPO-loaded scaffolds showing near-complete restoration by 8 weeks (Figure 5iiiH). Mechanistically, hypoxia-induced EPO expression via HIF-1 signaling contributed to enhanced bone regeneration. These findings establish rhEPO-loaded scaffolds as a promising strategy for bone defect repair. A key limitation of this study is the suboptimal method used for loading rhEPO into the PLGA scaffolds as simple soaking may not ensure sustained or controlled release, potentially affecting the consistency of *in vivo* results; additionally, although *in vitro* and *in vivo* models were employed, the underlying molecular mechanisms of EPO’s dual roles in angiogenesis and osteogenesis, particularly in relation to dose-dependency and signaling pathways, require further elucidation to optimize clinical application.

Steroid-induced avascular necrosis of the femoral head (SANFH) leads to osteoblast apoptosis, compromising bone integrity and hip function. Cai et al. (2023) explored EPO’s therapeutic potential in SANFH by targeting the STAT1-caspase 3 pathway. *In vitro*, EPO was non-toxic and reduced dexamethasone-induced apoptosis by downregulating BAX, cytochrome c, p-STAT1, and cleaved-caspase 3 and 9 while upregulating Bcl-2. In SANFH rat models, EPO decreased vacant bone lacunae, preserving bone structure, as confirmed by histology and micro-CT. This study has several limitations: a small sample size with potential variability among rats, incomplete understanding of EPO’s interaction with glucocorticoid receptor pathways, and uncertain applicability of rat model findings to human SANFH. The outcomes of this research highlight EPO’s role in mitigating bone loss via STAT1-caspase 3 inhibition, warranting further studies on dosing, long-term efficacy, and integration into bone tissue engineering strategies.

Beyond its role in erythropoiesis, EPO influences bone marrow stromal cells via its receptor on non-erythroid cells. Suresh et al. (2019) studied Tg6 mice with chronic EPO overexpression, showing increased hematocrit but reduced trabecular and cortical bone, fewer bone marrow adipocytes, and impaired BMP2-induced bone and fat formation. Short-term high-dose EPO treatment similarly elevated hematocrit while reducing bone mass and adipocytes and inhibiting BMP signaling without affecting osteoclasts. In ΔEpoRE mice, where EPO signaling is restricted to erythroid cells, trabecular bone was diminished, adipocytes increased, and ectopic bone formation was impaired. Notably, EPO treatment in these mice normalized hematocrit without bone loss, suggesting that EPO’s bone-reducing effects are independent of erythropoiesis. Transplantation experiments further revealed that Tg6 cells had impaired osteogenic and adipogenic potential, while ΔEpoRE cells favored fat formation over bone development. These findings underscore EPO’s critical role in bone marrow stromal cell differentiation, where dysregulated signaling disrupts the bone–fat balance and compromises skeletal homeostasis. The limitations of Suresh et al. (2019) include reliance on mouse models that may not fully translate to human physiology, discrepancies between *in vivo* and *in vitro* findings, and limited mechanistic insight into how EPO-EPOR signaling affects BMSC differentiation. Low EPOR expression in BMSCs raises questions about signaling relevance, and systemic inflammation in older ΔEpoRE mice may confound bone outcomes. Sex-based differences and the use of ectopic ossicle models also limit generalizability to normal bone physiology.

Reconstructing segmental bone defects is challenging due to limited self-healing capacity. Bone marrow mesenchymal stem cells (BMSCs) are promising for bone repair, but issues such as poor recruitment, limited differentiation, and potential tumorigenesis hinder their effectiveness. Li et al. (2019) demonstrated that EPO facilitates the migration of transplanted BMSCs to defect sites, promoting osteogenesis, angiogenesis, increased vessel density, callus development, and improved bone mineral density. The EPO + BMSC combination significantly strengthened diaphysis, reducing fragility. This study demonstrates the potential of EPO to mobilize transplanted BMSCs for bone defect repair but has several limitations. Notably, the absence of an EPO-only control group limits understanding of EPO’s independent effects. The use of a short-term, rodent-based model restricts translational relevance to human applications. Tumorigenic risk from BMSC proliferation, immune or inflammatory responses, and the effects of endogenous BMSCs were not evaluated. Additionally, no dose-response analysis was conducted for EPO, and long-term outcomes beyond 8 weeks remain unknown. Future research should optimize EPO dosing, explore long-term safety, and test its efficacy in clinical models to advance therapeutic applications.

### 4.2 Cartilage tissue engineering

Cartilage repair is inherently difficult due to the avascular nature of cartilage, which limits its natural healing capacity (Grande et al., 1999). Cartilage injuries remain a significant challenge, affecting millions worldwide and lacking a definitive cure. Stem cell transplantation has shown promise but is constrained by limited cell availability and safety risks. To overcome these limitations, a novel treatment using EPO-loaded hyaluronic acid (HA) micro-scaffolds (HA + EPO) has been developed. These micro-scaffolds demonstrated strong cell compatibility and targeted chondrocytes via CD44 receptors. The system was designed to gradually release EPO, reducing inflammation and recruiting progenitor cells to cartilage defects. The HA + EPO group exhibited a bone volume fraction of 66.54% ± 5.00% at 12 weeks and 85.30% ± 6.13% at 26 weeks, significantly higher than other groups. In a rabbit model of full-thickness cartilage defects, intra-articular injections of HA + EPO significantly reduced inflammatory cells in synovial fluid, outperforming other groups in both anti-inflammatory effects and progenitor cell recruitment. This led to enhanced chondrogenesis and effective cartilage repair. Unlike traditional stem cell therapies, this approach leverages localized growth factor delivery, offering a safer and more accessible alternative for treating cartilage injuries. Future studies will focus on refining scaffold performance, ensuring long-term efficacy, and advancing clinical translation (Hakamivala et al., 2020).

Osteoarthritis (OA), a leading cause of disability, frequently involves knee joint damage, with meniscal tears playing a critical role in its progression. Fu (2020) investigated the potential of enhancing meniscal regeneration to prevent OA. Developmental analysis of the mouse meniscus revealed active cell proliferation and apoptosis during embryonic and early postnatal stages, with Collagen I (Col-1) identified as a key structural component. EPO emerged as the most effective factor for promoting meniscal repair, increasing Col-1, Collagen II (Col-2), and VEGF-A expression while reducing MMP-13 levels in organ culture models. *In vivo*, EPO-treated mice showed improved meniscal repair and reduced OA progression in a meniscus injury-induced OA model. These findings highlight EPO’s potential as a disease-modifying osteoarthritis drug (DMOAD). Future studies will focus on optimizing dosage, exploring long-term outcomes, and evaluating EPO’s efficacy in larger animal models and clinical trials for early OA prevention.

### 4.3 Periodontal tissue engineering

Periodontitis, a chronic inflammatory condition that progressively destroys tooth-supporting structures, necessitates effective regenerative treatments. A notable advance in EPO delivery is the development of multifunctional hydrogel systems that not only enable localized and sustained release but also exhibit synergistic therapeutic effects. For instance, Gu et al. (2024) introduced a novel injectable thermosensitive hydrogel composed of EPO and FK506 embedded within a chitosan/β-glycerophosphate/gallic acid (EPO-FK506-CS/β-GP/GA) matrix for treating periodontitis. This formulation exhibited desirable physicochemical properties, including structural stability and controlled drug release. Upon application in a rat model, the hydrogel significantly downregulated inflammatory cytokines such as TNF-α, IL-6, and IL-1β, while promoting robust alveolar bone regeneration. Importantly, this system enhanced local osteogenesis, as evidenced by increased bone density in micro-CT scans and the upregulation of osteogenic markers such as Collagen I, Runx2, osteopontin (OPN), and osteocalcin (OCN). The incorporation of both EPO and FK506 allowed the hydrogel to leverage EPO’s angiogenic and osteogenic potential while modulating immune responses to prevent chronic inflammation. By delivering EPO in a sustained and localized manner, the hydrogel minimized systemic exposure and potential side effects, directly addressing the challenges of EPO’s short half-life and systemic toxicity. Promising avenues for future research include refining the hydrogel formulation, assessing long-term treatment outcomes, and conducting clinical trials to determine the efficacy and safety of this approach in human periodontal therapy.

**FIGURE 6:**
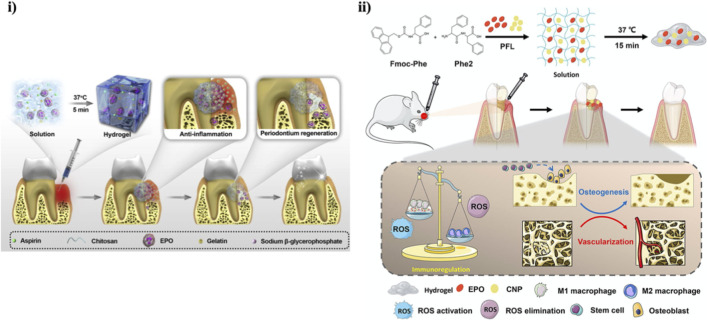
**(i)** Diagrammatic representation of the synthesis and application of hydrogels made of CS/β-GP/gelatin, reproduced with permission from Xu et al. (2019) (© 2019, Elsevier) **(ii)** Boosting periodontal regeneration through a novel injectable hydrogel system that enables the sequential release of nanoceria and EPO (Li et al., 2023).

To combat periodontitis, Xu et al. (2019) developed an injectable, thermosensitive hydrogel that combines chitosan (CS), β-sodium glycerophosphate (β-GP), and gelatin (Figure 6i. This innovative formulation aims to simultaneously diminish inflammation and facilitate the regeneration of periodontal tissues, providing a dual therapeutic approach for enhancing periodontal health. The hydrogel, loaded with aspirin for anti-inflammatory effect and EPO for tissue regeneration, demonstrated sustained drug release for 21 days and excellent biocompatibility both *in vitro* and *in vivo*. Micro-CT and immunohistochemistry confirmed its effectiveness in terminating inflammation and restoring alveolar bone height, while histological observations further validated its regenerative potential. The faster initial release of aspirin established an anti-inflammatory environment, enabling subsequent EPO-driven regeneration. These results suggest this hydrogel as a promising candidate for periodontitis treatment, with future efforts focusing on optimizing drug delivery, ensuring long-term efficacy, and advancing clinical translation.

Zheng et al. (2019) examined EPO’s influence on periodontal ligament stem cells (PDLSCs), focusing on how EPO affects the proliferation and osteogenic differentiation of these cells, as well as the underlying molecular mechanisms driving these processes. PDLSCs were isolated, characterized, and transfected with β-catenin shRNA to explore the involvement of the Wnt/β-catenin pathway. The application of EPO resulted in a minor decrease in PDLSC proliferation. However, it significantly enhanced osteogenic activity, as evidenced by increased alkaline phosphatase activity and calcium deposition (on day 21). Furthermore, EPO dose-dependently upregulated the expression of key osteogenic markers, including Runx2, Osterix, and OCN at both 7 and 14 days. It also upregulated β-catenin and cyclin D1 levels. However, β-catenin knockdown attenuated EPO’s osteogenic effects, suggesting that EPO promotes the osteogenic differentiation of PDLSCs by activating the Wnt/β-catenin signaling pathway. Future research will focus on optimizing EPO dosages, evaluating its long-term effects on periodontal regeneration, and validating these findings in *in vivo* and clinical models for therapeutic applications.

Periodontal disease often leads to the loss of alveolar bone and, eventually, teeth, with periodontal ligament stem cells (PDLSCs) being key to maintaining and repairing periodontal tissues. This study examined the role of EPO receptor (EPOR) in enhancing PDLSC-based therapies for periodontal regeneration. PDLSCs were isolated from individuals with chronic periodontal disease (PD-PDLSCs) and healthy donors (Cont-PDLSCs). PD-PDLSCs exhibited diminished regenerative capacity and lower EPOR expression than their healthy counterparts. Silencing EPOR in Cont-PDLSCs impaired their regenerative potential, mimicking the functionality of PD-PDLSCs, while the EPO-mediated activation of EPOR rejuvenated PD-PDLSCs' regenerative abilities. In a periodontitis mouse model, EPOR-activated PDLSCs effectively restored the periodontal ligament, cementum, and alveolar bone, while EPOR-silenced PDLSCs failed to do so. Furthermore, EPOR signaling in PDLSCs was shown to regulate immune suppression and maintain bone metabolism via osteoclast and osteoblast modulation. These findings underscore the importance of EPOR signaling in PDLSC-based regeneration, offering a promising direction for advanced periodontal therapies (Zakaria et al., 2024).

Bae et al. (2022) investigated the therapeutic potential of EPO in promoting bone regeneration and reducing inflammation in periodontitis models. *In vitro*, EPO at 10 IU/mL significantly increased human periodontal ligament fibroblast (hPDLF) proliferation approximately 1.3–1.4-fold at 24 and 48 h (*p* < 0.01), while MC3T3-E1 cells showed a 1.18- to 1.56-fold increase, with increased expression of osteogenic markers such as ALP, BMP-2, and osteocalcin over time. In an *in vivo* periodontitis model, local EPO application to extraction sockets significantly improved bone formation, as confirmed by histological analysis, and upregulated VEGF, CD31, RUNX2, and osteocalcin while reducing inflammatory markers such as myeloperoxidase. These findings suggest that EPO facilitates osteogenesis and mitigates inflammation, particularly in osteoblast-lineage cells. Future research aims to optimize EPO-based delivery systems, evaluate long-term outcomes, and explore clinical applications in periodontal regeneration.

To address the challenges of local drug delivery in irregular periodontal pockets caused by periodontitis, an injectable Fmoc-Phe3 hydrogel (FPH) loaded with ceria nanoparticles (CNP) and EPO (EPO/CNP@FPH) was developed by Li et al. (2023) (Figure 6ii). The hydrogel provided controlled release, with CNP released faster (69.67%) than EPO (61.33%) over 3 days, creating a regenerative microenvironment. By scavenging excess reactive oxygen species (ROS), the hydrogel facilitated a switch in macrophage polarization from the pro-inflammatory M1 to the anti-inflammatory M2 phenotype while also enhancing angiogenesis and osteogenesis. In a rat periodontitis model, the hydrogel demonstrated remarkable therapeutic efficacy, promoting irregular alveolar bone regeneration. These results highlight EPO/CNP@FPH as a promising candidate for periodontitis treatment. Future research will focus on optimizing the hydrogel composition, extending its application to other inflammatory bone diseases, and evaluating its long-term safety and effectiveness in clinical settings.

Angiogenic tissue engineering is critical for periodontal regeneration, and EPO, a multifunctional cytokine, has shown protective and angiogenic effects on periodontal ligament stem cells (PDLSCs). Huang et al. (2022) explored the role of EPO in inflammatory conditions by exposing PDLSCs to varying concentrations of tumor necrosis factor-α (TNF-α) and assessing EPO’s impact. Under inflammation, PDLSCs exhibited increased apoptosis, reduced autophagy, and impaired angiogenesis. Specifically, 20 IU/mL EPO significantly enhanced PDLSC proliferation, reduced pro-inflammatory cytokines (IL-1β and IL-8), downregulated the Bax/Bcl-2 ratio, and elevated angiogenic markers (VEGF-a, IGF-1, and FGF-2) and autophagy-related genes (Beclin1 and LC3B). These findings demonstrate that EPO protects PDLSCs from inflammatory damage and provides a promising foundation for advancing periodontal regeneration strategies.

### 4.4 Wound healing and skin regeneration

Wound healing requires the coordination of re-epithelialization, angiogenesis, and matrix remodeling (Yadav et al., 2025b), processes that EPO directly influences. Chronic wounds, such as diabetic ulcers and pressure sores, pose significant challenges due to impaired healing mechanisms. EPO-modified hydrogels and dressings have shown remarkable efficacy in enhancing keratinocyte migration, fibroblast activity, and vascularization in such wounds. By creating a pro-regenerative microenvironment, these constructs also reduce scarring by modulating extracellular matrix remodeling (Sorg et al., 2013).

Rionaldo et al. explored the effects of EPO on the viability of extended random skin flaps in a study involving 30 male Wistar rats divided into three groups. Flaps (2 cm × 8 cm) were treated with EPO injections of 50 IU or 100 IU, while the control group received no treatment. After 7 days, necrotic areas were measured, and capillary vessel density was analyzed using histopathology. Results showed a significant increase in capillary vessel density in both EPO-treated groups compared to controls (*p* = 0.000), with the 50 IU group demonstrating the most pronounced improvement in flap viability (*p* = 0.020). Interestingly, the 100 IU group did not significantly differ from controls in reducing necrotic areas (*p* = 0.680). These findings indicate that EPO, particularly at 50 IU, enhances flap viability through improved vascularization. Future research should focus on optimizing dosage, explore long-term outcomes, and assess EPO’s potential in clinical settings for wound healing and reconstructive surgery (Dhiparedja et al., 2023).

Diabetic foot ulcers (DFUs) are a serious complication of diabetes, often leading to substantial morbidity. Hamed et al. (2021) examined the potential of a topical EPO-containing hydrogel to enhance DFU healing. In a randomized, controlled trial, 20 patients meeting inclusion criteria were assigned to either daily topical EPO combined with standard-of-care (SOC) or SOC alone for 12 weeks. Weekly assessments tracked healing progress, with the primary goal being at least 75% ulcer closure. At 12 weeks, six out of ten patients in the EPO group achieved 75% closure, compared to only one out of eight in the SOC group. The average DFU area was significantly smaller in the EPO group (1.2 ± 1.4 cm^2^) compared to SOC alone (4.2 ± 3.4cm^2^; *p* = 0.023), with faster re-epithelialization in the EPO-treated ulcers. Importantly, no adverse effects were observed. These results highlight topical EPO as a promising solution for DFU management. Future research should focus on broader trials, long-term outcomes, and integrating this approach into routine care.

Adipose tissue-derived microvascular fragments (MVFs) are valuable for vascularization in tissue engineering; Später et al. (2021) demonstrated the potential of systemic low-dose EPO to enhance their performance. MVFs isolated from donor mice were seeded onto collagen-glycosaminoglycan matrices and implanted into full-thickness skin defects in recipient mice treated daily with either EPO (500 IU/kg) or saline. EPO treatment reduced endothelial cell apoptosis (cleaved caspase-3+) while maintaining proliferation (Ki67+), leading to faster and more effective vascularization, improved blood vessel maturation, and enhanced epithelialization *in vivo*. These findings highlight EPO’s capacity to boost MVF viability and vascular network formation, offering a new avenue to optimize vascularization strategies in tissue engineering. Future research should focus on determining the optimal EPO dosing regimen, assess long-term vascular stability, and translate this approach to clinically relevant models to advance its application in regenerative medicine.

Papic et al. (2024) investigated the impacts of rhEPO and/or mineral trioxide aggregate (MTA) on inflamed dental pulp in a Wistar rat model. Inflammation was induced in the maxillary first molars (n = 64), with treatments including rhEPO, MTA, MTA + rhEPO, or an inert membrane, with healthy controls for comparison. After 4 weeks, histological and molecular analyses revealed that rhEPO and MTA + rhEPO significantly reduced inflammation and promoted mineralization. The MTA + rhEPO group exhibited notably lower levels of pro-inflammatory cytokines (TNF-α and IL-1β) and higher dentin matrix protein 1 expression, while both rhEPO and MTA + rhEPO treatments enhanced transforming growth factor-beta 1 levels and improved antioxidant markers. These results suggest that rhEPO, either alone or combined with MTA, offers favorable or superior outcomes compared to MTA alone, highlighting its potential as an advanced pulp capping material for managing inflamed dental pulp.

Ngo et al. (2024) introduced an innovative hemostatic dressing for open wounds, combining chitosan (CS), silk fibroin (SF), and montmorillonite (MMT). The CSSF@MMT dressing addresses key challenges in wound care by enhancing hemostasis, providing antimicrobial action, and supporting angiogenesis while maintaining optimal moisture levels. The dressing demonstrated impressive mechanical strength and rapid hemostatic properties. When loaded with ciprofloxacin (CIP), it exhibited sustained drug release over a week (around 93%) and effective antibacterial activity against both Gram-positive and -negative bacteria. EPO-loaded CSSF@MMT dressings showed promising results in promoting tissue regeneration. *In vitro* cell migration assays revealed stimulated endothelial cell proliferation and migration, while chick embryo chorioallantoic membrane studies (vascular area increased by ∼2.3 ± 0.2-fold from day 7–14 with CSSF@MMT@EPO, compared to ∼1.7-fold in controls) confirmed significant vascular regeneration. The research suggests that CSSF@MMT dressings, incorporating CIP and EPO, offer a multifaceted approach to wound care. They effectively stop bleeding, reduce inflammation, create a protective environment, and promote tissue regeneration. This advanced dressing could potentially reduce the need for limb amputations and alleviate the global burden of wound care. Future research will focus on optimizing the dressing formulation, assess its long-term effects on wound healing, and conduct clinical trials to validate its efficacy across various wound care applications. These advances in wound management technology hold promise for improving patient outcomes and reducing healthcare costs associated with chronic wound care.

Burn wound trials have been more promising: a 2018 Phase II study (EudraCT 2006-002886–38) reported that EPO accelerated re-epithelialization by 30% and reduced sepsis incidence in severe burns, likely via VEGF-driven angiogenesis and reduced inflammation (Günter et al., 2018). Mechanistically, EPO enhanced VEGF-driven angiogenesis and collagen deposition, correlating with improved granulation tissue formation and reduced inflammation, as confirmed by post-hoc histological analyses. These findings align with a 2021 systematic review affirming that topical/subcutaneous EPO accelerates wound healing via faster re-epithelialization, anti-inflammatory effects, and macrophage polarization toward pro-regenerative M2 phenotypes (Toleubayev et al., 2021).

### 4.5 Cardiac and vascular repair

Cardiac and vascular tissues are highly sensitive to ischemia and poor perfusion (Saludas et al., 2017), making EPO an ideal candidate for therapeutic intervention. EPO-modified hydrogels have been developed as cardiac patches to deliver localized doses of EPO to infarcted myocardium (Kobayashi et al., 2008). These patches reduce apoptosis in cardiomyocytes, stimulate angiogenesis, and improve overall left ventricular function in preclinical models. In vascular engineering, EPO-modified grafts enhance endothelialization and reduce thrombosis, improving the integration and long-term performance of vascular grafts (Lin et al., 2011). These constructs are especially relevant in treating myocardial infarctions, peripheral artery disease, and vascular bypass surgeries.

EPO has roles beyond hematopoiesis, including cytoprotection, inotropy, and neurogenesis, but its physiological relevance in the heart remained unclear. Allwood et al. (2024) showed that cardiac EPO mRNA followed a distinct circadian rhythm in adult mice and increased during embryogenesis, suggesting its physiological importance throughout life. Using cardiomyocyte-specific EPO knockout mice (EPOΔ/Δ-CM), it was found that the loss of EPO production in cardiomyocytes led to reduced EPO expression and cellular proliferation during cardiogenesis, but in adults, heart weight was preserved through the hypertrophy of cardiomyocytes. There was actually a 600-fold increase in cardiac EPO mRNA expression and a six-fold increase in EPO expression in enriched neonatal cardiomyocytes relative to controls. Although cardiac dimensions did not change, echocardiography revealed a small decline in ejection fraction, stroke volume, and tachycardia, while invasive hemodynamics showed increased contractility. Interestingly, EPO expression in adult hearts was elevated, particularly in endothelial cells, which were identified as the source of this increased expression. These findings suggested that EPO in cardiomyocytes is essential for regulating cardiac function, and the loss of production in these cells triggered compensatory EPO expression from other cells. Future research will focus on further understanding the molecular mechanisms behind EPO expression shifts in cardiac cell types and exploring its therapeutic potential for heart disease.

Klopsch et al. (2018) comparing the effects of epicardial, intraperitoneal, and intramyocardial EPO treatments following acute myocardial infarction (MI) in rats. Their research aimed to address the global health burden of ischemic heart failure by exploring ways to enhance intracardiac regenerative mechanisms. Their study, involving 156 rats, found that epicardial EPO administration (300 U kg−1) was the most effective method for stimulating regenerative processes after MI. Within 24 h post-MI, epicardial EPO significantly increased the expression of key regenerative markers, including SDF-1, CXCR4, CD34, Bcl-2, cyclin D1, Cdc2, and MMP2. It also activated TGF-β/Wnt signaling pathways in intramyocardial MSC niches and promoted cell proliferation. Six weeks after MI, cardiac catheterization and tissue analysis revealed improved cardiac function, beneficial remodeling, and enhanced capillary density in rats treated with epicardial EPO. Further investigations using various techniques, including fluorescence-activated cell sorting, co-cultures with neonatal cardiomyocytes, and angiogenesis assays, demonstrated that EPO stimulated cardiomyogenic differentiation and paracrine angiogenesis activity in cardiac MSCs (CD45^−^CD44+DDR2+). The findings of Klopsch et al. (2018) suggest that epicardial EPO administration effectively upregulates regenerative signals after MI. This early stimulation of mesenchymal proliferation, combined with synergistic angiogenesis and direct activation of TGF-β/Wnt signaling in cardiac MSCs, accelerates the healing process and improves cardiac recovery. These results offer promising insights into potential therapeutic strategies for enhancing cardiac regeneration and recovery following myocardial infarction.

The therapeutic vascular conduit (TVC) addresses the challenge of inadequate vascularization in cell-based therapies by using a decellularized vessel scaffold surrounded by a hydrogel sheath containing protein-secreting cells. This design allows the TVC to be directly anastomosed as a vascular graft. Computational models suggested that the TVC facilitates oxygenated blood flow near transplanted cells, preventing hypoxia. In a proof-of-principle study using EPO, Han et al. (2021) implanted TVCs in nude rats, which increased serum EPO and hemoglobin levels for up to 4 weeks. Nude rats implanted with EPO-TVCs exhibited sustained plasma EPO levels of ∼20 mIU/mL and elevated hemoglobin (Hgb) from 15 g/dL to 20 g/dL, maintained for up to 4 weeks. Subcutaneous implants delivered a transient burst of EPO (up to 15 mIU/mL on day 1), with minimal levels by day 7. However, continuous EPO expression caused macrophage infiltration and luminal obstruction, limiting its long-term efficacy. Further studies indicated that EPO may also recruit macrophages, complicating its use. While the TVC shows potential for overcoming oxygenation barriers in large-scale cellular implantation, these findings emphasize the need to understand the biological effects of therapeutic proteins. Future research should focus on regulating protein expression, exploring alternative therapeutic proteins with fewer side effects, preventing luminal obstruction, and evaluating the TVC’s durability and safety over time. Additionally, its application in treating other clinical conditions warrants further investigation to advance this technology toward clinical use.

In cardiac repair, the EPICURE trial (2024) evaluated EPO combined with iron in anemic patients undergoing transcatheter aortic valve implantation (TAVI). While EPO failed to reduce red cell transfusions (RR 0.77, *p* = 0.63), it showed non-significant trends in lowering morbidity (SOFA scores) (Urena et al., 2017). In contrast, the REPAIR-AMI trial (2006) demonstrated that intracoronary EPO post-myocardial infarction improved left ventricular ejection fraction (LVEF) by 5.5% at 4 months, though later trials such as HEBE III reported no benefit due to high thromboembolic risks with systemic dosing (Schächinger et al., 2006).

### 4.6 Neural tissue repair

Neural tissue engineering seeks to address the limited regenerative capacity of neurons following injury or degeneration (Pramanik et al., 2023b). In neural tissue repair, EPO has been shown to exert neuroprotective and tissue-repair effects that are mechanistically distinct from its classical erythropoietic function, particularly in models of ischemic, inflammatory, and traumatic neural injury. Experimental work associated with the Cerami laboratory demonstrated that these neuroprotective actions are not mediated by the canonical erythroid EPOR homodimer but instead involve a distinct receptor system activated under conditions of cellular stress and injury (Brines et al., 2004). This receptor system has been described as a heteromeric EPOR–β-common receptor (βcR/CD131) complex, often referred to as the “tissue-protective” or “innate repair” receptor, which is upregulated in neuronal and glial cells following hypoxia or inflammatory insult (Brines and Cerami, 2008). Importantly, non-erythropoietic EPO derivatives such as carbamylated EPO (CEPO) were shown to retain neuroprotective and anti-apoptotic activity while lacking hematopoietic effects, providing functional evidence for receptor-selective signaling in neural repair (Leis, 2004).

EPO-modified scaffolds have been broadly investigated for their neuroprotective effects, including the prevention of neuronal apoptosis, reduction of oxidative stress, and promotion of axon regeneration (Hernández et al., 2017). In spinal cord injury models, EPO-modified constructs support the survival and differentiation of neural stem cells, facilitating functional recovery. Similarly, EPO-loaded hydrogels have shown promise in stroke models by enhancing neurogenesis and protecting against ischemic damage (Gorio et al., 2002; Kontogeorgakos et al., 2009; Carelli et al., 2011; Matis and Birbilis, 2009). These applications are being expanded to neurodegenerative diseases, such as Alzheimer’s and Parkinson’s, where EPO could slow disease progression and promote repair.

**FIGURE 7:**
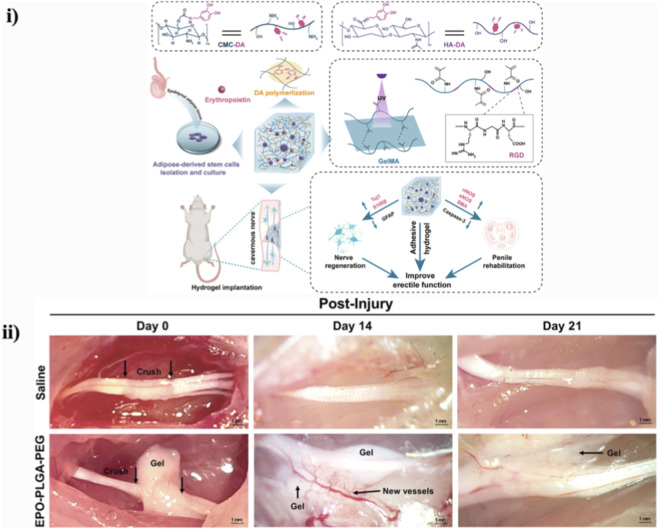
**(i)** GelMA hydrogel was fabricated and its adhesiveness enhanced via dopamine polymerization. The EPO-loaded hydrogel was combined with ADSCs and applied to bilateral cavernous nerve crush injury sites to restore erectile function through nerve regeneration and penile rehabilitation (Shao et al., 2022). **(ii)** Images show EPO-PLGA-PEG biodegradation after application to a 3 mm sciatic nerve crush site. At 14 days post-injury, abundant blood vessels formed a microvascular network around the injection area. The gel was still present on the nerve at day 21, demonstrating prolonged retention. These findings were consistent across five subjects per group, with images scaled to a 1 mm bar (Manto et al., 2022).

Neurogenic erectile dysfunction (nED), a persistent postoperative complication following rectal and prostate cancer surgeries, has been addressed through an innovative approach combining stem cell therapy with a multifunctional hydrogel. Shao et al. (2022) developed an EPO-loaded hydrogel enhanced with catechol-catechol adducts (Figure 7i), thus improving its adhesive properties and mechanical strength. The hydrogel achieved a swelling ratio of 60.3%, adhesive strength of ∼9 kPa (3× higher than GelMA alone), and a Young’s modulus of ∼400 kPa for pure GelMA under 50% compression. EPO release showed an initial burst, with ∼70% released within 2 days. This formulation significantly enhanced adipose-derived stem cell (ADSC) retention and viability at the injury site while promoting Schwann cell migration and PC12 cell differentiation *in vivo*. When tested on a bilateral cavernous nerve injury rat model, the stem cell-EPO-hydrogel implantation strategy demonstrated significant alleviation of erectile dysfunction, accelerated neural differentiation, and suppressed astrocyte development in the major pelvic ganglia. The therapy also restored expression levels of key proteins in penile tissues and preserved vascular endothelium content while preventing penile fibrosis. These promising results suggest that this combined approach could revolutionize both nED treatment and clinical translation of stem cell therapy. Future research should improve the hydrogel’s composition, investigate long-term effects and safety, explore applications in other neurological disorders, conduct larger-scale preclinical studies, and develop strategies for clinical implementation.

Le et al. (2022) explored EPO-loaded nanobots (ENBs) as a targeted delivery system for neuroprotection in central nervous system injuries. ENBs, developed using nanospray drying, demonstrated enhanced EPO release after preconditioning sonication—at 50–60 kHz for 1 h, this significantly enhanced erythropoietin (EPO) release from ENBs, with cumulative release reaching 39.2% at 4 h, 61.7% at 12 h, and 84.1% at 24 h, compared to only 8.4%, 21.2%, and 25.1%, respectively, without sonication. *In vitro* experiments with SH-SY5Y neuronal cells showed that both EPO and ENBs provided comparable neuroprotection against thapsigargin-induced stress, maintaining cell viability above 65% and exhibiting similar expression patterns of pro-apoptotic and apoptotic markers. These findings suggest that sonication-preconditioned ENBs can effectively control EPO release and provide neuroprotection equivalent to free EPO. Future research should focus on *in vivo* studies to validate these results, optimize ENB formulations for extended release, investigate potential side effects, and explore applications in various neurological disorders. Additionally, developing strategies for targeted delivery to specific brain regions and combining ENBs with other neuroprotective agents could further enhance their therapeutic potential.

In the ongoing quest for effective treatments for neurological disorders, researchers have shifted their focus to spinal cord injury (SCI), a devastating condition with limited therapeutic options. Leveraging the principles of controlled drug delivery systems, a team of scientists explored the potential of EPO-loaded hydrogels as a novel approach to SCI treatment. Gholami et al. (2021) investigated the local therapeutic effects of EPO-chitosan/alginate (EPO-CH/AL) hydrogels on the apoptotic and inflammatory indices associated with SCI secondary injury. The researchers fabricated EPO-CH/AL hydrogels using ionic gelation and characterized them with SEM and FT-IR. They demonstrated that EPO-CH/AL hydrogels provided sustained EPO release over 240 h, with cumulative release reaching 76%, 87%, and 93% for the 1000, 5000, and 10,000 IU/kg formulations, respectively. The hydrogels were tested in a rat SCI model using various EPO doses. All EPO-CH/AL hydrogels significantly reduced inflammation and apoptosis indices in SCI-inflicted rats. However, the 1000 IU/kg EPO dose showed the most promising results, significantly improving tissue repair and histopathological appearance at injury sites. The study suggested that EPO-CH/AL hydrogels, particularly at 1000 IU/kg EPO, can effectively improve experimental SCI in rats by inhibiting apoptosis and inflammation. Future research should elucidate the scaffold’s role in the observed effects, optimize hydrogel composition for enhanced biocompatibility, investigate long-term outcomes, explore combination therapies with other neuroprotective agents, and conduct larger-scale preclinical studies to validate efficacy and safety for potential clinical translation in SCI treatment.

Similarly, in a groundbreaking study, Rasekh et al. (2025) explored the potential of EPO/alginate/chitosan (CH-AL) hydrogel as a novel treatment for SCI-induced male infertility. Using a rat model, they compared three groups: sham, SCI-induced without intervention, and SCI-induced treated with 10,000 IU/kg EPO-CH/AL hydrogel. The results were promising, showing significant improvements in sperm viability and motility, reduction in oxidative stress markers, and mitigation of apoptosis in the treatment group. Histological analysis revealed partial restoration of germ cell population and luminal spaces. These findings suggest that EPO-CH/AL hydrogel could be a potent therapeutic strategy for addressing SCI-induced infertility. Future research should concentrate on optimizing the hydrogel formulation, investigating long-term effects, exploring combination therapies with other fertility-enhancing agents, and conducting larger-scale preclinical studies to validate efficacy and safety. Additionally, translational studies in human subjects and the development of targeted delivery systems could pave the way for clinical applications in treating SCI-induced male infertility.

Shan et al. (2024) investigated the role of EPO in regulating astrocyte pyroptosis following spinal cord injury (SCI) using integrated bioinformatic and experimental approaches. Analysis of the GEO dataset GSE153720 revealed the enrichment of pyroptosis-related genes in astrocytes after SCI, primarily associated with the NLRP3 inflammasome pathway. Both *in vivo* and *in vitro* experiments demonstrated that EPO significantly reduced astrocyte pyroptosis. Mechanistically, EPO was shown to upregulate miR-325-3p in astrocytes, leading to the suppression of gasdermin D (Gsdmd), a key executor of pyroptosis. The inhibition of miR-325-3p reversed the EPO-mediated downregulation of Gsdmd, confirming the functional relevance of the miR-325-3p/Gsdmd axis. These findings identify a specific molecular pathway through which EPO exerts neuroprotective effects in SCI and provide a mechanistic basis for its therapeutic potential in spinal cord repair.

In the ongoing search for effective treatments for neurological disorders, researchers have developed a novel EPO-derived peptide, DEPO, that targets vascular dementia. Zhou et al. (2024) evaluated DEPO’s safety, erythropoiesis effects, and neuroprotective properties in both *in vitro* and *in vivo* models. Using naive C57BL6 mice and a bilateral common carotid artery stenosis (BCAS) mouse model, they found that DEPO did not significantly affect hemoglobin or red blood cell counts after 4 weeks of treatment, unlike rhEPO, which increased hemoglobin by ∼30%. However, they demonstrated significant neuroprotective effects, alleviating spatial reference memory impairment and anxiety levels in BCAS mice, while reducing both gray and white matter injuries. Mechanistically, DEPO activated the JAK/STAT pathway in cultured neurons, protecting them against chronic ischemia through the EPOR and its downstream signaling. These findings suggest that DEPO is a safe and potentially effective treatment for vascular dementia, warranting further investigation into its long-term effects, optimal formulation, and potential combination therapies. Future research should focus on larger-scale preclinical and clinical studies to validate DEPO’s efficacy and safety for clinical translation in vascular dementia treatment.

Manto et al. (2022) explored the potential of EPO encapsulated in amphiphilic PLGA-PEG block copolymers as a localized, sustained-release therapeutic for traumatic peripheral nerve injury (TPNI). The EPO-PLGA-PEG formulation demonstrated remarkable physiochemical properties, transitioning from solution to gel within a physiologically relevant temperature range. It maintained stable EPO release for over 2 weeks (approx. 18 days) with an initial burst release of ∼26.5% (0.5 IU/µL) on day 1 and cumulative release reaching 83.6% by day 12. When applied to a murine sciatic nerve crush injury model, the treatment yielded significant improvements in functional recovery metrics, including sciatic function index, grip strength, and withdrawal reflex. Moreover, the therapy enhanced neurovascular regeneration, evidenced by increased blood vessel density, vascular junctions, increased myelinated axon percentage (91.7% vs. 75.3% in saline group), and myelinated nerve fibers in the injured area. As shown in Figure 7ii, the gel was applied to a 3 mm crush injury site. By day 14, a rich network of blood vessels had formed at the injection site, indicated by arrows. In contrast, saline and vehicle-treated animals showed minimal vascularization. The gel remained present on the nerve at day 21, demonstrating adherence and controlled degradation. These promising preclinical results suggest that EPO-PLGA-PEG could be a valuable addition to TPNI treatment strategies. Future research should seek to optimize the formulation for extended release periods, investigate its efficacy in larger animal models, and explore its potential in treating other types of nerve injuries. Additionally, studies examining the long-term safety profile, dose-response relationships, and combination with other regenerative therapies could further advance this approach towards clinical translation.

For neural regeneration, Neuro-EPO—a low-sialic acid EPO variant—achieved neuroprotection without erythropoietic effects in Alzheimer’s models, reducing Aβ plaques by 40% and improving cognition in transgenic mice. A Phase II trial (NCT04318314) is currently testing intranasal Neuro-EPO in early Alzheimer’s patients, leveraging its BBB penetration and minimal systemic exposure (Rama et al., 2019).

## 5 Emerging applications in biomedical research and therapy

**FIGURE 8:**
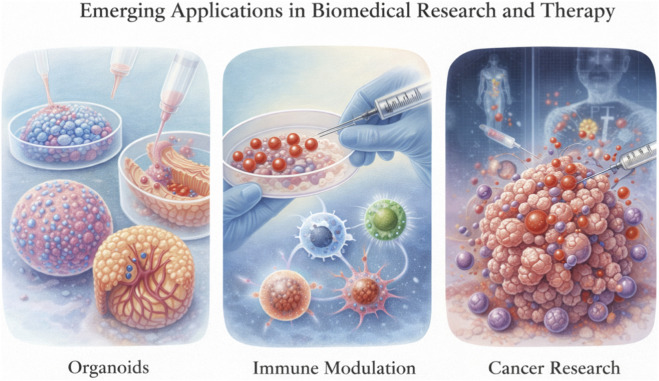
Emerging applications of EPO-modified systems in biomedical research and therapy, highlighting their use in organoid models, immune modulation, and cancer research.

EPO-modified systems are increasingly applied in organoid models, immune modulation, and cancer research to study disease mechanisms and improve therapeutic strategies (Figure 8).

### 5.1 Organoids

Organoids are 3D cellular structures that resemble the architecture and function of specific organs (Lou and Leung, 2018). Organoid systems represent advanced *ex vivo* tissue-engineering platforms that recapitulate key aspects of tissue architecture, cellular heterogeneity, and microenvironmental signaling. Within this context, the use of erythropoietin and EPO-modified constructs in organoid models provides a mechanistically relevant framework for studying tissue repair, regeneration, and immune-modulatory responses in a controlled, translational setting. EPO-modified constructs have been integrated into organoid cultures to support cell survival and differentiation (Eliopoulos et al., 2003). For instance, liver and kidney organoids enhanced with EPO demonstrate improved vascularization and functional maturation, enabling more accurate modeling of tissue repair processes (Yang and Lewis, 2020; Tsujimoto et al., 2024). These systems can also be used for drug discovery, where EPO-modified organoids provide platforms for testing the efficacy and safety of novel therapeutics (Tebon et al., 2023). Recent studies have demonstrated that EPO contributes significantly to organoid viability, growth, and lineage specification, particularly under stress or injury-mimicking conditions (Eliopoulos et al., 2003), (Harbuzariu et al., 2019). In neural organoids, EPO has been shown to enhance neuroepithelial expansion and promote neuronal differentiation by activating signaling cascades such as JAK2/STAT5 and PI3K/AKT (Hirano and Suzuki, 2019; Suzuki et al., 2013). These pathways not only support the survival of neural progenitors but also improve synaptogenesis and axonal integrity, leading to more physiologically relevant brain organoid models. For example, in models of hypoxic injury (Pan et al., 2023), EPO treatment reduced cell death and enhanced neural network activity, indicating its neuroprotective utility during early developmental stages. EPO promotes neurogenesis and oligodendrocyte maturation by activating EPOR isoforms expressed in neural cells (Rey et al., 2019). In rodent models, EPO enhances astrocyte proliferation and oligodendrocyte survival—mechanisms critical for neural organoid maturation. This neuroprotective effect could improve the viability of patient-derived neural organoids for modeling neurodegenerative diseases such as Alzheimer’s (Roberto de Barros et al., 2023). While EPO is not explicitly studied in cardiac organoids, its pro-angiogenic properties such as inducing endothelial progenitor mobilization, activating STAT5 signaling in endothelial cells, promoting capillary formation, and enhancing VEGF expression in bone marrow stromal cells could address the vascularization challenges in heart-forming organoids (HFOs), which currently lack mature vasculature despite containing endothelial precursors (Tsiftsoglou, 2021; Drakhlis et al., 2021). Its pro-angiogenic effects mediated via upregulation of VEGF and mobilization of endothelial progenitor cells could facilitate improved perfusion-like conditions within organoids—critical for the long-term viability and maturation of myocardial tissue constructs (Trillhaase et al., 2021). Furthermore, EPO has been used in liver organoid cultures to promote hepatic progenitor cell proliferation and increase albumin production, cytochrome P450 expression, and urea synthesis, all of which are markers of hepatic functional maturity (Yang and Lewis, 2020). These effects are thought to result from the EPO-mediated modulation of Wnt/β-catenin and ERK1/2 pathways, which are central to liver development and regeneration. In renal organoids, EPO is primarily produced by tubular epithelial cells and glomerular mesangial cells under hypoxic conditions. It exhibits renoprotective effects by inhibiting apoptosis and reducing interstitial fibrosis—crucial for modeling chronic kidney disease (CKD). Additionally, EPO has been shown to attenuate epithelial-to-mesenchymal transition (EMT), a key process in renal fibrosis progression, thereby improving organoid functionality and effectively mimicking pathological states (Ding Beichen et al., 2020; Du et al., 2024; Zhang et al., 2020). In bone organoids, EPO stimulates osteoblast proliferation and differentiation through JAK-STAT signaling pathways. It also promotes the production of BMPs, such as BMP2 and BMP6, which enhance osteogenesis and cartilage formation. However, prolonged exposure to recombinant human EPO (rHu-EPO) can lead to increased bone turnover and trabecular bone loss, highlighting the need for controlled application in organoid-based studies. These findings suggest EPO’s utility in understanding skeletal regeneration and bone homeostasis (Nadezhdina et al., 2024; Lappin et al., 2021; McGee et al., 2012). The inclusion of EPO in microphysiological systems bridges the gap between *in vitro* and *in vivo* studies, offering a versatile tool for translational research.

Du et al. (2024) developed kidney organoids that overexpress erythropoietin (EPO+) by using a non-integrating DNA vector with a scaffold/matrix attachment region (S/MAR) containing the EPO gene. Their study indicated that S/MAR DNA vectors effectively maintained transgene expression throughout the differentiation process of kidney organoids. This finding suggests that these vectors are capable of ensuring stable genetic activity during the various stages of organoid development. EPO + organoids showed increased podocyte and endothelial cell markers, with a reduction in distal tubular cell markers. Subcutaneous implantation of EPO-expressing organoids in immunocompromised mice yielded intriguing results. After a month, these organoids maintained higher EPO mRNA expression and showed a marked increase in endothelial cell population over the control group. Critically, the study revealed significant physiological effects in mice implanted with EPO + organoids and a correlation between the number of implanted organoids and blood hematocrit levels. As the quantity of organoids increased, so did the hematocrit levels in the recipient mice. Additionally, Du et al. (2024) observed a striking enlargement of these animals’ spleens. EPO + organoids were found to enhance the expression of fibroblast growth factor 23 (FGF23) mRNA, which stimulates osteoblast activity, in the bone marrow. They also influenced the composition of trabecular bone. These results highlight the promise of gene-edited kidney organoids as a potential tool for endocrine restorative therapies. Future research should focus on optimizing the S/MAR DNA vector design, investigate long-term effects and safety profiles, explore the potential for treating other kidney-related endocrine disorders, and conduct larger-scale preclinical studies. Additionally, developing strategies for targeted organoid implantation and assessing the integration of these organoids with the host kidney tissue could further advance this promising approach towards clinical application in treating kidney-disease-related anemia.

Ding B. et al. (2020) developed a novel method for generating three-dimensional (3D) renal organoids from whole kidney cells, offering a suitable tool for drug screening and potential regenerative therapies. The researchers optimized the initial cell concentration (8000 cells/well) and culture period (14 days) for optimal organoid formation and viability. These multicellular organoids expressed key renal cell markers, involving glomerular (podocin, synaptopodin, and nephrin), proximal tubule (AQP1), and collecting duct (AQP3) markers, indicating the presence of multiple renal cell types. Importantly, the organoids demonstrated the ability to produce EPO under hypoxic conditions, a crucial function of kidney cells. EPO production was time-dependent, oxygen-dependent, and only detected in first-passage renal cells. The organoids also showed potential as a tool for nephrotoxicity screening, responding to known nephrotoxic drugs such as aspirin, penicillin G, and cisplatin. These findings suggest that the 3D renal organoids developed by Ding B. et al. (2020) have the potential to serve as a valuable tool for drug screening, nephrotoxicity testing, and possibly as a model for studying kidney diseases, offering advantages over traditional 2D cell cultures and animal models.

Kim et al. (2023) adopted an innovative approach to generate erythroid lineage cells from human endometrium-derived induced pluripotent stem cells (iPSCs). Their protocol employed a two-phase culture system, with the second phase critically incorporating recombinant EPO alongside other factors to drive erythroid differentiation. The researchers utilized discarded endometrial tissues from hysterectomy patients to create iPSCs through electrotransfection with specific episomal vectors. The initial phase involved an 8-day culture to promote hematopoietic stem cell commitment, while the subsequent 22-day feeder-free phase included EPO as a key component to induce erythroid differentiation. EPO’s role in this protocol was crucial, as it is a primary regulator of erythropoiesis. Its inclusion in the culture medium likely stimulated the proliferation and maturation of erythroid progenitors, contributing to the high yield of erythroblasts observed. The researchers reported a stable yield of approximately 80% polychromatic and orthochromatic normoblasts, indicating successful erythroid differentiation. This optimized protocol demonstrates the potential of endometrium-derived iPSCs as a source for erythroid lineage cells, expanding the possibilities for tissue regeneration using human iPSC lines. The success of Kim et al. (2023) in directing erythroid differentiation from these cells opens new avenues for research in hematology and regenerative medicine.

Asal et al. (2024) investigated the role of EPO in small intestine models. They compared epithelial (EPI) models to full thickness (FT) models containing stromal cells. Key findings showed that EPO secretion was higher in EPI models than FT models. EPO levels inversely correlated with transepithelial electrical resistance (TEER) values, with FT models showing lower TEER and EPO secretion. The decreased EPO levels in FT models corresponded with reduced TEER and increased zonulin expression, indicating increased permeability. EPO has been shown to have a protective effect on the intestinal barrier, helping to maintain its integrity. Additionally, it has been observed to restore normal levels of ZO-1 expression, a protein crucial for tight junction formation in the intestinal epithelium. The hydrogel substrate and stromal cells in FT models appeared to influence EPO secretion and barrier properties more than the epithelial cells alone. Organoid-derived FT models showed TEER values closer to physiological levels than cell line models. These findings highlight EPO’s role in regulating intestinal barrier function and permeability, demonstrating the importance of including stromal components when developing physiologically relevant intestinal models. Future research could explore optimizing EPO levels in FT models to achieve more *in-vivo*-like barrier properties.

Collectively, these findings suggest that EPO not only supports organoid survival under challenging *in vitro* conditions but also actively modulates lineage-specific differentiation and functional maturation. This has profound implications for disease modelling, particularly of ischemic, neurodegenerative, and fibrotic disorders, as EPO-treated organoids exhibit more robust tissue-specific responses and improved structural integrity. Additionally, EPO’s role in reducing inflammation and apoptosis within organoid systems enhances relevance for drug screening and toxicity testing. In the context of personalized medicine, combining patient-derived induced pluripotent stem cells (iPSCs) with EPO-based organoid culture protocols may potentially generate tailored tissue models for predicting therapeutic outcomes, testing individual drug responses, and even autologous transplantation. Therefore, incorporating EPO into organoid platforms represents a highly promising strategy to bridge the gap between basic developmental biology and translational regenerative medicine.

### 5.2 Immune modulation applications

The immunomodulatory properties of EPO have been explored to develop constructs that reduce inflammation and improve implant biocompatibility. Biomaterials that include EPO have demonstrated the ability to inhibit the production of inflammatory mediators such as TNF-α and IL-6. This reduction in pro-inflammatory cytokines creates conditions more conducive to tissue repair and regeneration (Todosenko et al., 2016). These constructs are particularly useful in autoimmune conditions and in preventing implant rejection. For instance, EPO-modified scaffolds have demonstrated reduced immune activation, highlighting their potential in immunoengineering (Zhou et al., 2017).

EPO exerts significant anti-inflammatory effects on monocytes and macrophages, which abundantly express the EPO receptor. In murine models, EPO suppresses the production of inflammatory mediators such as TNFα, IL-6, and iNOS by inhibiting NF-κB pathways. Notably, EPO-treated mice infected with *Salmonella typhimurium* showed reduced inflammatory cytokine expression but increased bacterial burden compared to controls. Similarly, human monocytes exposed to EPO demonstrate decreased IL-6 and IL-8 production when stimulated with TLR ligands. These findings highlight EPO’s potent immunomodulatory role, particularly in dampening inflammatory responses in monocytes and macrophages, which may have implications for both infectious and inflammatory conditions (Nairz et al., 2011).

Paolo and colleagues conducted a study that revealed the significant immunomodulatory effects of EPO on human T-cell alloimmunity. Their research revealed that both CD4^+^ and CD8^+^ T cells express EPO receptors (EPO-R) on their surfaces. EPO was found to induce a dose-dependent reduction in allogeneic CD4^+^ T-cell proliferation without causing cell death. This effect required direct signaling through EPO-R on T cells and resulted in decreased Th1 differentiation. Mechanistically, EPO prevented IL-2-induced proliferation by disrupting IL-2 receptor signaling and inhibiting the phosphorylation of AKT and extracellular signal-regulated kinase. The researchers verified these effects *in vivo* using a mouse model, where EPO treatment led to reduced expansion of human naïve CD4^+^ T cells. Interestingly, while activated T cells expressed CD131 (an alternative EPO receptor), a specific CD131 agonist did not alter T-cell proliferation or cytokine production. These findings establish a link between EPO-R signaling on T cells and the inhibition of T-cell immunity, potentially explaining the protective effects of EPO observed in kidney transplant recipients. This research provides new insights into the immunomodulatory properties of EPO and its potential applications in transplant medicine and other T cell-mediated conditions (Cravedi et al., 2014).

Purroy et al. (2017) revealed the significant immunomodulatory properties of EPO in kidney transplantation and its role in promoting graft acceptance. They discovered that EPO stimulated antigen-presenting cells to secrete active TGF-β, which subsequently facilitated the conversion of naïve CD4^+^ T cells into functional Foxp3+ regulatory T cells (Tregs). Studies in mouse models revealed that pharmacologically reducing kidney-produced EPO inhibited the spontaneous formation of Tregs. Conversely, administering recombinant EPO extended the survival of heart allografts in these animals. Clinically, EPO administration increased the frequency of peripheral CD4^+^CD25^+^CD127lo T cells in humans with chronic kidney disease. This study also demonstrated that EPO directly inhibited conventional T cell proliferation while facilitating Treg proliferation through differential effects on IL-2 receptor signaling. This research highlights EPO’s ability to promote immune tolerance and suggests that modulating the EPO/EPO receptor signaling pathway could potentially be used to prevent or treat conditions driven by T cell-mediated immune responses, such as organ transplant rejection. Future research should focus on optimizing EPO-based therapies for enhancing transplant outcomes, develop targeted EPO derivatives that maximize immunomodulatory effects while minimizing erythropoietic side effects, and explore the potential of EPO in treating autoimmune diseases and other T cell-mediated disorders.

EPO has demonstrated therapeutic potential in various central nervous system disorders, but its clinical application is limited by hematocrit elevation. To address this, researchers developed EPO-derived peptides, hypothesizing that the molecule’s erythropoietic and tissue-protective functions reside in different regions. The 19-mer JM-4 peptide, derived from the Aβ loop of EPO, emerged as a potent immunomodulator in experimental autoimmune encephalomyelitis models without affecting hematocrit levels. JM-4 exhibited remarkable immunoregulatory properties, mirroring whole-molecule EPO’s effects on both peripheral and central nervous system inflammation. The treatment inhibited the function of monocytes and dendritic cells in antigen presentation, while also decreasing the number of T helper 17 cells. It led to a reduction in the production of inflammatory cytokines and simultaneously increased the population of regulatory T-cells. Future research should focus on elucidating JM-4’s molecular mechanisms, investigating its efficacy in other inflammatory conditions, conducting preclinical safety studies, exploring synergistic effects with existing therapies, and developing targeted delivery systems. This study opens new avenues for harnessing EPO’s therapeutic potential while minimizing side effects, potentially revolutionizing the treatment of inflammatory disorders affecting the central nervous system and beyond (Yuan et al., 2015).

### 5.3 Cancer research models

EPO’s role in erythropoiesis and angiogenesis has made it a valuable tool in cancer research. EPO-modified constructs are used to model the tumor microenvironment, providing insights into tumor-associated erythropoiesis and vascularization. These models are particularly useful for studying how cancer cells influence surrounding tissues and for testing the effects of anti-angiogenic therapies (Hardee et al., 2006; Hedley et al., 2011a; Hedley et al., 2011b). Although the therapeutic use of EPO in cancer patients remains controversial due to its potential to promote angiogenesis, its applications in preclinical research are advancing our understanding of tumor biology.

The integration of EPO-modified constructs in regenerative medicine offers transformative solutions for a wide range of clinical challenges. By directly addressing critical issues such as poor vascularization, inflammation, and limited cellular survival, these constructs enhance tissue repair in diverse applications, from bone and cartilage regeneration to cardiac and neural repair.

Bessoles et al. (2024) investigated the immunosuppressive effects of EPO in a mouse model of triple-negative breast cancer, shedding light on potential risks associated with EPO treatment in cancer patients. They found that EPO administration promoted tumor growth and exacerbated the “immune desert” phenomenon, resulting in a “cold tumor” environment. EPO altered immune cell distribution in peripheral blood, secondary lymphoid organs, and the tumor microenvironment (TME). Notably, EPO primarily affected CD4 T cells, accelerating their activation in the spleen and subsequent exhaustion in the TME. This process was accompanied by increased CD39 expression across various immune cells, particularly CD4 T cells in the tumor and spleen, contributing to an immunosuppressive TME. Bessoles et al. (2024) also identified a highly immunosuppressive CD39^+^ regulatory T cell population (ICOS+, CTLA4+, and Ki67+) as a potential biomarker for EPO-induced tumor progression risk. These findings highlight EPO’s pleiotropic immunosuppressive functions and its role in enhancing mammary tumor progression in mice. Future research should focus on validating these results in human cancer patients, explore potential strategies to mitigate EPO’s immunosuppressive effects, investigate the molecular mechanisms underlying EPO’s impact on CD4 T cells, and develop targeted approaches to counteract the immunosuppressive TME induced by EPO treatment.

Juvenile myelomonocytic leukemia (JMML) is a rare but fatal blood cancer, with leukemogenic SHP2 mutations present in 35% of patients. Despite its severity, JMML research has been hampered by a lack of representative cell models, which are crucial for investigating the disease’s pathogenesis and developing new therapeutic approaches. To address this critical gap, Zhao et al. (2023) developed innovative cell models that mimic SHP2-mutant JMML. They created stable cell lines by introducing SHP2-D61Y/E76K mutations into HCD-57 cells—a murine erythroleukemia line normally dependent on EPO for survival. These modified cells exhibited several key characteristics of JMML, including EPO-independent survival and proliferation, activated MAPK signaling consistent with SHP2-mutant JMML, and sensitivity to dasatinib and trametinib—drugs known to inhibit SHP2-mutant JMML cells. Importantly, when injected into immune-deficient mice, the mutant SHP2-transformed HCD-57 cells rapidly proliferated in the spleen and bone marrow. This *in vivo* model provides an excellent platform for testing potential therapies to target aberrant SHP2 signaling. The newly established HCD-57/SHP2-E76K and -D61Y cell lines, dependent on mutant SHP2 signaling for survival, closely resemble SHP2-mutant JMML. These models offer valuable tools for exploring the pathogenic mechanisms of mutant SHP2 and evaluating targeted therapies for SHP2-mutant JMML, thus addressing a critical need in the field of hematological malignancy research.

ShujaaEdin et al. (2021) investigated the impacts of rHuEPO on MCF-7 breast cancer cells in both two- (2D) and 3-dimensional (3D) cultures. Their results revealed that rHuEPO significantly reduced the viability of MCF-7 cells in 3D cultures more effectively than in 2D cultures, in a dose- and time-dependent manner. It downregulated caspase activity and induced a cytostatic effect by causing cells to enter the subG0/G1 phase of the cell cycle. These findings suggest that rHuEPO modulates cancer cell behavior differently in 3D environments, which better mimic *in vivo* conditions. Future research should focus on exploring the underlying mechanisms of rHuEPO’s cytostatic effects, assess its impact on other cancer cell lines, and investigate its potential therapeutic applications or risks when used alongside chemotherapeutic agents in cancer treatment.

Beh et al. (2019) engineered an innovative drug delivery system dubbed “EPO-TAMNLC” which combines tamoxifen-loaded nanostructured lipid carriers with an erythropoietin coating. This novel approach aimed to amplify tamoxifen’s cancer-fighting prowess and homing ability by exploiting EPO as a targeting mechanism for EPO receptors (EpoRs) prevalent on breast cancer cells. The team pitted EPO-TAMNLC against its uncoated counterpart, TAMNLC, using LA7 cells and LA7-induced rat mammary tumors as test subjects. Immunocytochemical analysis revealed the presence of both estrogen receptors (ERs) and EpoRs on LA7 cells. *In vitro* experiments demonstrated that EPO-TAMNLC and TAMNLC both significantly curbed LA7 cell proliferation, with effects intensifying as dosage and exposure time increased. Notably, EPO-TAMNLC triggered apoptosis and halted cell cycle progression at the G0/G1 phase. The *in vivo* trials showcased the tumor-suppressing capabilities of both formulations. Remarkably, intravenous administration of either compound at 5 mg/kg body weight proved non-toxic in rats, underscoring their potential as safe therapeutic agents. The researchers concluded that EPO-TAMNLC represents a groundbreaking dual-action drug delivery platform that can precisely target breast cancer tissues expressing both ERs and EpoRs. The integration of tamoxifen into these nanocarriers, with or without the EPO coating, markedly enhanced the specificity and safety profile of the remedy for mammary gland tumors.

## 6 Current status of EPO-related therapeutics

While EPO has been historically developed and approved for the management of anemia, its well-established clinical safety profile and pleiotropic biological functions have stimulated growing interest in applications beyond erythropoiesis. In recent years, EPO and EPO-modulating agents have been increasingly explored in tissue engineering and regenerative medicine, including organoid systems, immune modulation, and cancer-related models, where EPO signaling influences angiogenesis, cell survival, inflammation, and microenvironmental remodeling. Concurrently, the therapeutic landscape has expanded from conventional erythropoiesis-stimulating agents (ESAs) to include hypoxia-inducible factor prolyl hydroxylase (HIF-PH) inhibitors that enhance endogenous EPO production. To contextualize these emerging applications within a translational framework, Table 1 summarizes the current regulatory and developmental status of major EPO-related drugs, highlighting approved agents, compounds in advanced clinical development, and discontinued candidates relevant to future bioengineering and disease-modeling strategies.

**TABLE 1:** Clinically relevant EPO-related therapeutics with translational relevance to tissue engineering and emerging applications.

Drug (INN)	Class/EPO relevance	Regulatory status (selected regions)	Relevance to tissue engineering and emerging applications	Reference
Epoetin alfa	Recombinant human EPO (ESA)	Approved (US, EU)	Benchmark EPO molecule widely used in angiogenesis, cell survival, organoid culture, and immune modulation studies due to its well-characterized biology and clinical safety.	fda and cder (2025)
Darbepoetin alfa	Long-acting EPO analog (ESA)	Approved (US, EU)	Extended half-life enables sustained EPO signaling, relevant for long-term tissue-engineered constructs and chronic *in vitro* models.	fda and cder (2025)
Methoxy polyethylene glycol–epoetin beta (CERA)	Long-acting ESA	Approved (US, EU)	Provides prolonged EPO receptor activation; attractive for chronic stimulation in engineered tissues and vascularized constructs.	CHMP (2025)
Epoetin alfa biosimilars (e.g., epoetin alfa-epbx)	ESA (biosimilar)	Approved (US, EU)	Cost-effective and scalable EPO sources facilitating broader translational and experimental use in tissue engineering research.	fda and cder (2025)
Vadadustat	HIF-PH inhibitor → increases endogenous EPO	Approved (US; dialysis-dependent CKD)	Enables indirect EPO activation via HIF stabilization, relevant for immune–vascular crosstalk in engineered tissues.	fda and cder (2025)
Roxadustat	HIF-PH inhibitor → increases endogenous EPO	Approved (Japan, China); not approved (US/EU)	Extensively studied HIF–EPO modulator; valuable in organoid, ischemia, and cancer microenvironment models despite regional regulatory variability.	Martínez-Miguel et al. (2025)
Enarodustat	HIF-PH inhibitor → increases endogenous EPO	Approved (Japan)	Represents next-generation endogenous EPO modulation; relevant for controlled hypoxic signaling in advanced *in vitro* platforms.	Fukui et al. (2021)
Desidustat	HIF-PH inhibitor → increases endogenous EPO	Approved (India)	Emerging agent of interest for studying EPO-linked angiogenic and immune effects in engineered tissues and disease models.	Dhillon (2022)

## 7 Clinical translation of EPO-based tissue engineering strategies

Despite the expanding clinical availability of EPO-related therapeutics, the direct translation of EPO into tissue-engineered constructs remains largely preclinical. Nevertheless, EPO has been evaluated in several clinical studies that address tissue repair and regeneration, including wound healing, ischemic injury, and neuroprotection—contexts that share fundamental biological principles with tissue engineering approaches. These clinical investigations provide valuable insights into dosing, safety, and the regenerative efficacy of EPO beyond erythropoiesis, thereby informing the design of future engineered tissues and advanced *in vitro* models.

To clarify the translational progress of EPO-based tissue engineering strategies, Table 2 summarizes reported tissue-engineering-related applications of EPO and indicates whether these approaches have advanced to clinical evaluation, including available ClinicalTrials.gov identifiers. Collectively, these data highlight a substantial translational gap between preclinical innovation and the clinical implementation of EPO-functionalized engineered tissues, underscoring key opportunities for future research.

**TABLE 2:** Tissue engineering-relevant applications of EPO with translational/clinical status (https://clinicaltrials.gov/).

Application	Role of EPO	Clinical trial (NCT) and translational status
Neuroprotection/brain injury	Promotes neurogenesis, reduces apoptosis, and supports vascularization and metabolic adaptation	NCT00987454, completed
Premature neonatal neuroprotection	Supports neural survival in pre-term infants at risk of brain injury	NCT01378273, completed
Topical EPO for diabetic wounds	Suppression of apoptosis, promotion of angiogenesis and repair	NCT02361931, completed
Hypoxic-ischemic encephalopathy (HIE)	Combined EPO and hypothermia to reduce brain injury	NCT02811263, completed
EPO in systemic injury/ischemia	Investigated for tissue preservation and repair pathways	NCT00425698, completed
Skin tissue engineering/wound healing	Angiogenesis, re-epithelialization, cytoprotection	NCT00212219, completed
Burn wound repair	Vascularization, tissue survival	NCT00656152, completed
Bone tissue engineering/fracture healing	Coupled angiogenesis–osteogenesis	NCT00952289, early clinical exploration

## 8 Conclusion and future prospects

EPO has developed from being principally recognized for its hematopoietic function to becoming a versatile biomolecule with diverse implications in tissue engineering and regenerative medicine. This review has emphasized EPO’s pleiotropic effects, comprising its pro-angiogenic, anti-apoptotic, neuroprotective, and immunomodulatory characteristics, which make it a crucial candidate for supporting tissue repair and regeneration. The successful integration of EPO into tissue-engineered structures has yielded substantial advances across a spectrum of applications, including bone, cartilage, neural, cardiac, dental, wound healing, and skin tissue engineering, as well as in emerging biomedical fields such as organoid technology, immune modulation, and cancer research. These findings emphasize EPO’s potential to revolutionize regenerative strategies, though the journey toward clinical application remains fraught with challenges.

A vital challenge lies in optimizing the delivery systems for EPO to ensure precise, sustained, and localized release while preserving its bioactivity. Methods such as scaffold immobilization, encapsulation, and gene therapy approaches have demonstrated promise but often require further refinement to overcome issues such as rapid degradation, diffusion into non-target tissues, and loss of functionality. Moreover, achieving the ideal therapeutic dose of EPO without triggering systemic side effects, such as erythropoiesis or off-target angiogenesis, is essential for its safe application in clinical settings. Another substantial obstacle is the scalability and reproducibility of EPO-based constructs for widespread use. Developing economical, robust manufacturing methods that adhere to stringent regulatory standards is imperative. Moreover, concerns related to the immunogenicity of recombinant EPO and its derivatives must be addressed to diminish the risk of adverse reactions, especially in long-term therapies. EPO’s complex role in cancer biology poses another layer of difficulty. While EPO has demonstrated immunomodulatory and protective effects in regenerative applications, its potential to foster tumor progression or interfere with cancer treatments necessitates a deeper understanding of its dual roles to harness its benefits without unintended consequences.

EPO-based therapies have demonstrated significant potential in promoting bone regeneration, yet their transition from laboratory research to clinical application remains complex. While preclinical studies have shown promising results in enhancing osteogenesis, translating these findings into clinical practice requires multiple challenges to be addressed, including regulatory approvals, long-term stability, and safety considerations. The integration of EPO into biomaterial scaffolds introduces additional regulatory complexities. Biomaterials intended for clinical use must comply with stringent safety and efficacy requirements established by regulatory agencies such as the FDA and EU’s Medical Device Regulation (MDR). The MDR classifies advanced therapies such as EPO-impregnated scaffolds as combination products, subject to dual oversight under both medical device and medicinal product regulations, which increases development costs by ∼30% and extends timelines by 12–18 months (Jurczak et al., 2025; Berishvili et al., 2024). The combination of a biologically active molecule such as EPO with a synthetic or natural scaffold often places these constructs in the category of combination products, necessitating rigorous preclinical and clinical evaluations. Standardizing manufacturing processes, ensuring batch-to-batch consistency, and validating the biocompatibility of EPO-loaded scaffolds are critical steps in gaining regulatory approval. A major consideration for the clinical use of EPO-based biomaterials is their long-term stability. The controlled and sustained release of EPO from scaffolds is essential for maintaining therapeutic efficacy, yet degradation kinetics and bioactivity retention remain significant challenges. Factors such as scaffold composition, degradation profile, and storage conditions influence the structural integrity and functional performance of the EPO-loaded construct. The optimization of carrier materials, encapsulation techniques, and sterilization methods are necessary to ensure prolonged bioactivity without compromising scaffold integrity. The long-term stability of EPO-loaded scaffolds, while demonstrated in preclinical settings (Fayed et al., 2024), lacks human trial validation, raising concerns about polymer degradation kinetics and sustained bioactivity beyond 6 months. Safety risks remain paramount: systemic EPO use increases thromboembolism and stroke risks (Ma et al., 2022; Åberg et al., 2016; Lippi Massimo and Favaloro Emmanuel, 2010; Anaissie et al., 2012). These complications are largely attributed to elevated hematocrit levels and increased blood viscosity resulting from excessive erythropoiesis, particularly in patients receiving high or sustained doses of EPO. Additionally, EPO has been associated with hypertension, possibly due to vasoconstriction and increased peripheral vascular resistance mediated by EPO receptor activation on endothelial and smooth muscle cells. EPO-induced hypertension affects 10%–30% of patients, peaking within 2–4 weeks of therapy initiation (Brar et al., 2024). Localized delivery via scaffolds may mitigate systemic toxicity but still risks unintended VEGF-driven angiogenesis in tumors. Despite its regenerative potential, the clinical use of EPO-loaded scaffolds must account for potential side effects. EPO has been associated with off-target effects, including erythropoiesis stimulation, increased risk of thrombosis, and potential tumorigenic concerns under certain conditions. Localized delivery strategies, such as controlled-release formulations or tissue-specific targeting, may help mitigate these risks by limiting systemic exposure. Additionally, long-term *in vivo* studies are essential to evaluate immune responses, unintended vascularization, and any adverse effects associated with scaffold degradation products.

Looking toward the future, the integration of cutting-edge technologies is poised to address these challenges. Advances in biomaterial science are expected to yield scaffolds with improved biocompatibility and tunable properties that allow for the controlled, sustained release of EPO. Personalized medicine approaches, including 3D bioprinting and patient-specific cell therapies, could further enhance the efficacy and specificity of EPO-based treatments. Additionally, the incorporation of EPO in organoid research provides a promising avenue for studying its effects in complex, physiologically-relevant systems, paving the way for breakthroughs in organ regeneration and disease modeling. The utilization of hypoxia-controlled bioreactors to mimic physiological oxygen gradients will enhance endogenous EPO production and its effects on organoid development. Moreover, the integration of microfluidic systems to simulate perfusion and nutrient exchange will improve the relevance of EPO-treated organoids for disease modeling. CRISPR/Cas9 can be used to create organoids with hypoxia-inducible EPO expression systems, enabling autocrine regulation of growth factors. The use of EPO in immune modulation also holds significant promise, particularly in conditions such as autoimmune diseases or chronic inflammation, where its immunoregulatory effects could be leveraged to restore homeostasis. In cancer research, a dual focus on understanding EPO’s tumor-promoting and anti-tumor effects may lead to innovative therapies that exploit its benefits while mitigating risks. Novel formulations and delivery systems such as tissue-targeted nanoparticles, hydrogel-based scaffolds, and gene-controlled EPO expression are under investigation to achieve localized effects while minimizing systemic exposure. More in-depth mechanistic studies are needed to elucidate the context-specific effects of EPO in various tissue types, particularly in relation to dose, timing, and receptor subtype involvement. Developers are adopting “safe-by-design” strategies, such as covalent EPO conjugation to polymers such as Eudragit EPO to prevent leakage and leveraging modular manufacturing platforms to streamline regulatory compliance. Currently, combination therapies such as antiplatelet agents or ACE inhibitors are explored to counteract thrombotic and hypertensive effects. Monitoring protocols for thrombosis markers and iron levels are also essential for safe administration. Additionally, further investigation into non-erythropoietic EPO analogs or derivatives may provide safer therapeutic alternatives with reduced hematopoietic activity. Finally, integrating multi-omics approaches and machine learning models could help identify patient-specific responses and biomarkers, thus advancing the use of EPO in precision medicine. To fully realize the potential of EPO in tissue engineering and beyond, continued multidisciplinary collaboration will be essential. Researchers in molecular biology, materials science, and clinical medicine must work together to address the remaining hurdles, including dose optimization, long-term safety, and regulatory compliance. Furthermore, robust preclinical and clinical trials will be critical to translating experimental successes into tangible therapeutic options.

In conclusion, EPO is a transformative biomolecule with the potential to redefine the field of regenerative medicine. Its applications in tissue engineering, organoid development, immune modulation, and cancer therapy highlight its versatility and promise. By overcoming the existing challenges and leveraging emerging technological advancements, EPO-based interventions have the capacity to address some of the most pressing medical challenges, offering new hope for patients and advancing the frontiers of biomedical science.
